# Investigating the impacts of heavy metal(loid)s on ecology and human health in the lower basin of Hungary’s Danube River: A Python and Monte Carlo simulation-based study

**DOI:** 10.1007/s10653-023-01769-4

**Published:** 2023-10-16

**Authors:** Omar Saeed, András Székács, Győző Jordán, Mária Mörtl, Mostafa R. Abukhadra, Mohamed Hamdy Eid

**Affiliations:** 1 Doctoral School of Environmental Science, Hungarian University of Agriculture and Life Sciences (MATE), Páter Károly u. 1, Gödöllő, 2100 Hungary; 2 Agro-Environmental Research Centre, Institute of Environmental Sciences, Hungarian University of Agriculture and Life Sciences, Herman Ottó út 15, Budapest, H-1022 Hungary; 3 Eötvös Loránd University (ELTE), Budapest, Hungary; 4 Geology Department, Faculty of Science, Beni-Suef University, Beni-Suef, 65211 Egypt; 5 Institute of Environmental Management, Faculty of Earth Science, University of Miskolc, Miskolc, 3515 Hungary

**Keywords:** Heavy metals, Metal indices, Human health and ecological risks, Monte Carlo simulation, Sensitivity analysis

## Abstract

This study aimed to determine the environmental and health risks of the heavy metal levels in the Danube River in Hungary. The metals, including Fe, Mn, Zn, Cu, Ni, Cr, Pb, and As, were measured in the period from 2013 to 2019. The Spearman correlation and heatmap cluster analysis were utilized to determine the origin of pollution and the factors that control surface water quality. Several indices, such as the heavy metal pollution index (HPI), metal index (MI), hazard quotient oral and dermal (HQ), hazard index oral and dermal (HI), and carcinogenic risk (CR), were conducted to evaluate the potential risks for the environment and human health. The values of the HPI were between the range of 15 < HPI < 30, which indicated moderate pollution; however, the MI results showed high pollution in Dunaföldvár and Hercegszántó cities. The ecological risk (RI < 30) and HI values (< 1) showed low environmental risks and non-carcinogenic impacts of the existing metals, either on adults or children. The mean CR value of oral arsenic was 2.2E−04 and 2.5E−04 during April–September and October–March, respectively, indicating that children were the most vulnerable to arsenic-carcinogenic oral effects. While lead’s CR oral values for children during April–September exceeded the threshold of 1.0E−04, chromium’s oral and dermal CR values for both adults and children were 2.08E−04, 6.11E−04, 1.97E−04, and 5.82E−04 during April–September and October–March, respectively. These results demonstrate the potential carcinogenic risks related to chromium exposure within the two pathways in Hungary and highlight the need for effective measures to mitigate these risks.

**Supplementary Information:**

The online version contains supplementary material available at 10.1007/s10653-023-01769-4.

## Introduction

The presence of heavy metals in aquatic environments has raised significant global concerns due to their potential adverse effects on human health (Ali et al., 2016; Chowdhury et al., 2016). These heavy metals are recognized as systemic toxins capable of causing damage to various organs and leading to teratogenic and carcinogenic effects (Tchounwou et al., 2012). Some metals, such as copper (Cu), zinc (Zn), iron (Fe), and manganese (Mn), are essential for human metabolism at certain levels but can become toxic when their concentrations exceed the permissible limits of drinking water. On the other hand, metals like arsenic (As) and lead (Pb) have no physiological role and can disrupt the endocrine system. Heavy metals can find their way into the human body through many pathways, including oral consumption, dermal contact, and inhalation (Jafari et al., 2019; Mohammadi et al., 2019; Rezaei et al., 2019). These elements are significant contaminants in different drinking water resources, such as surface water and groundwater (Izah et al., 2016), beverages (Izah et al., 2017), and vegetables (Chibueze Izah & Omozemoje Aigberua, 2017). Additionally, heavy metals are known to be prevalent air pollutants (Uzoekwe & Ajayi, 2018; Di Vaio et al., 2018). Their introduction into the environment is primarily attributed to industrial, agricultural, domestic, medical, and technological activities, as well as the use of materials holding these metals (Tchounwou et al., 2012). Excessive levels of heavy metals in drinking water, exceeding the permissible limits set by international organizations, can lead to acute and chronic health problems. These health issues range from less severe circumstances like muscle weakness to more serious illnesses, for example, nervous system dysfunction, brain disorders, and cancer. To guarantee the protection of the environment and human health, it is important to examine water quality. The primary step involves assessing the overall water quality and then detecting the source of pollutants to mitigate pollution levels. A well-established technique to evaluate water quality with respect to heavy metals is the heavy metal pollution index (HPI), HQ, and HI integrated with the Monte Carlo method. Furthermore, cluster analysis has proven to be an effective tool for classifying the sources accountable for presenting heavy metals into surface water and groundwater. Using these approaches offers a reliable understanding and representation of the condition of the water body. This, in turn, helps in developing a comprehensive management plan intended at reducing pollution levels (Sethi et al., 2023). Potential health risk assessment involves the calculation of chronic daily intake and relevant absorption coefficients to quantitatively estimate potential human health risks associated with heavy metal exposure (Li et al., 2014; Yang et al., 2015). To comprehensively assess the ecological risk posed to the environment, researchers have utilized various indices, including the heavy metal pollution index (HPI), the metal index (MI), and the potential ecological risk index (RI) (Hakanson, 1980; Venkata Mohan et al., 1996). To enhance analysis efficiency, additional techniques have been employed, such as multivariate statistical analyses aimed at identifying potential sources of heavy metals (Race et al., 2015). Moreover, geographic information system (GIS) methods have been applied to examine spatial pollutant distribution patterns and determine potential pollutant sources (Tiwari et al., 2015, 2016). Addressing the challenge of parameter uncertainty and obtaining accurate results, the Monte Carlo simulation method has gained popularity in assessing potential health risks associated with toxic substances present in surface water, groundwater, and soil (Sheng et al., 2021; Shokoohi et al., 2021). In light of this, the current research study employs the Monte Carlo simulation technique to conduct a robust assessment of probabilistic health risks linked to hazardous materials (HMs) in the surface water of the lower Danube River basin in Hungary. This approach ensures a thorough evaluation by accounting for uncertainties and providing a more accurate representation of potential health risks posed by heavy metal contamination.

The Danube River, a remarkable aquatic ecosystem, fosters an astonishing variety of plant and animal life. However, the looming specter of heavy metal pollution casts a shadow over its thriving diversity. Research has uncovered that heavy metals, when present in aquatic environments, can reverberate across an extended timeframe, leaving an indelible impact on various denizens of these waters. This impact extends to fish populations (ichthyofauna), the myriad of organisms inhabiting the water’s bed (benthic fauna), and the lush aquatic flora (macrophytes). This recognition has galvanized scientists to direct their efforts toward both mitigating pollution sources and curtailing the deleterious effects of heavy metals on these aquatic organisms. A central tenet of their endeavors underscores the critical role of consistent monitoring of pollutants within aquatic ecosystems, facilitated through diverse analytical methods (Calmuc et al., 2021; Calmuc et al., 2020; Sambito and Freni, 2021). The formidable sources of heavy metal pollution within the confines of the Lower Danube region encompass a constellation of influences. Sewage discharge, municipal waste, fertilizers, pesticides, the combustion of fossil fuels, and a spectrum of activities tethered to navigation and mining converge as primary contributors (Calmuc et al., 2021). In light of these concerns, the potential risks wrought by trace metals for the inhabitants and beneficiaries of the Danube River cannot be dismissed lightly. As this iconic river traverses through the territories of nine of Europe’s most industrially prolific nations, a clarion call emerges for an augmented, systematic regimen of heavy metal monitoring and assessment. These actions, rooted in regularity and meticulousness, are paramount to safeguarding the integrity and sustainability of the aquatic realm within the expansive bounds of the Danube River region.

The aims of this study were to carry out a thorough analysis of the potential risks posed by heavy metals on both the environment and human health in the lower basin of the Danube River watershed in Hungary; to (i) identify potential sources of the heavy metals by employing multivariate statistical techniques such as Spearman correlation analysis and heatmap cluster analysis along with the interpolation IDW technique; (ii) assess the potential non-carcinogenic and carcinogenic human health risks from heavy metals; and (iii) apply the Monte Carlo method to decrease the uncertainty and predict the values of hazard quotients (HQ) for heavy metals.

**Fig. 1:**
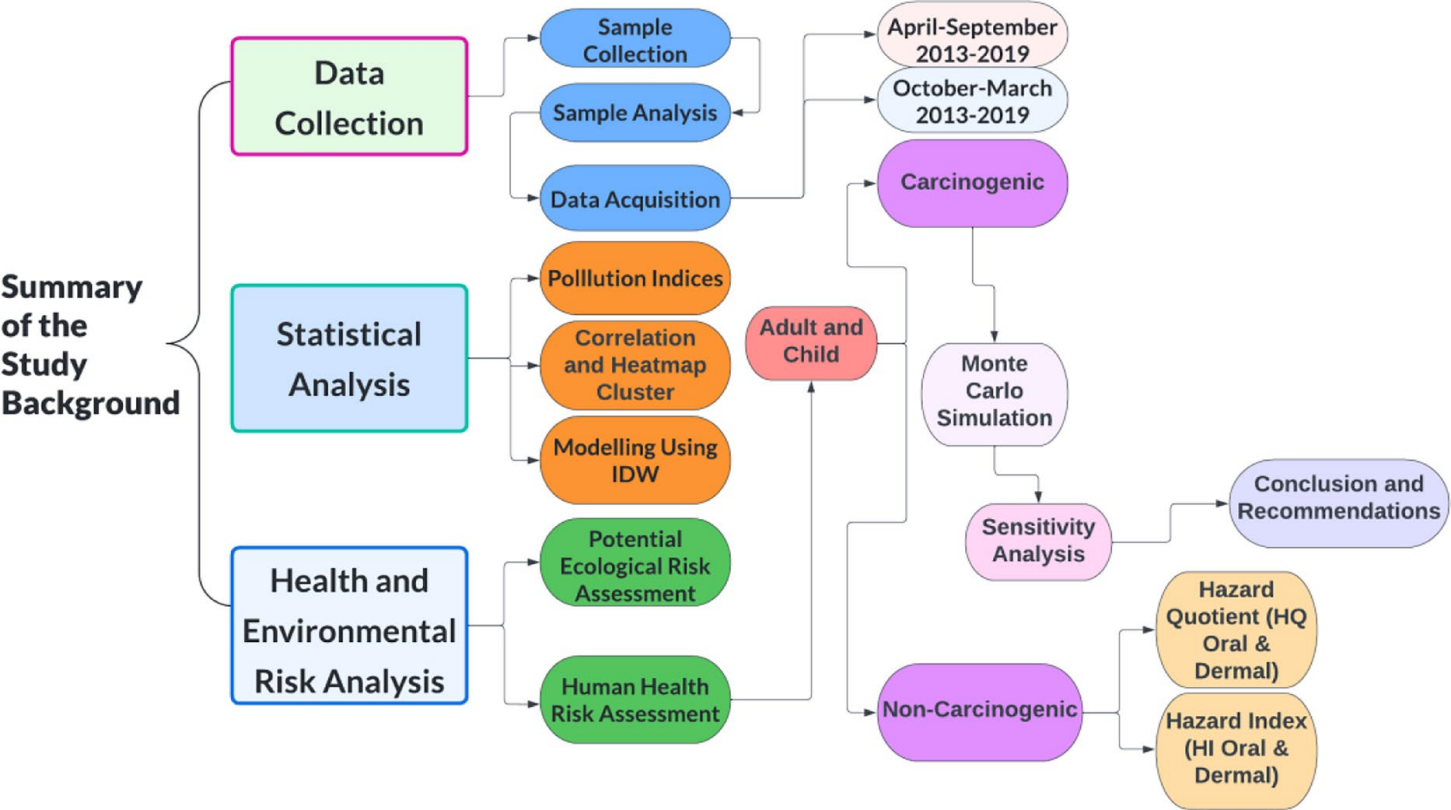
Flowchart of the current research

The flowchart of the current research was presented schematically in Fig. 1.

## Materials and methods

### Study area description and water sampling

**Fig. 2:**
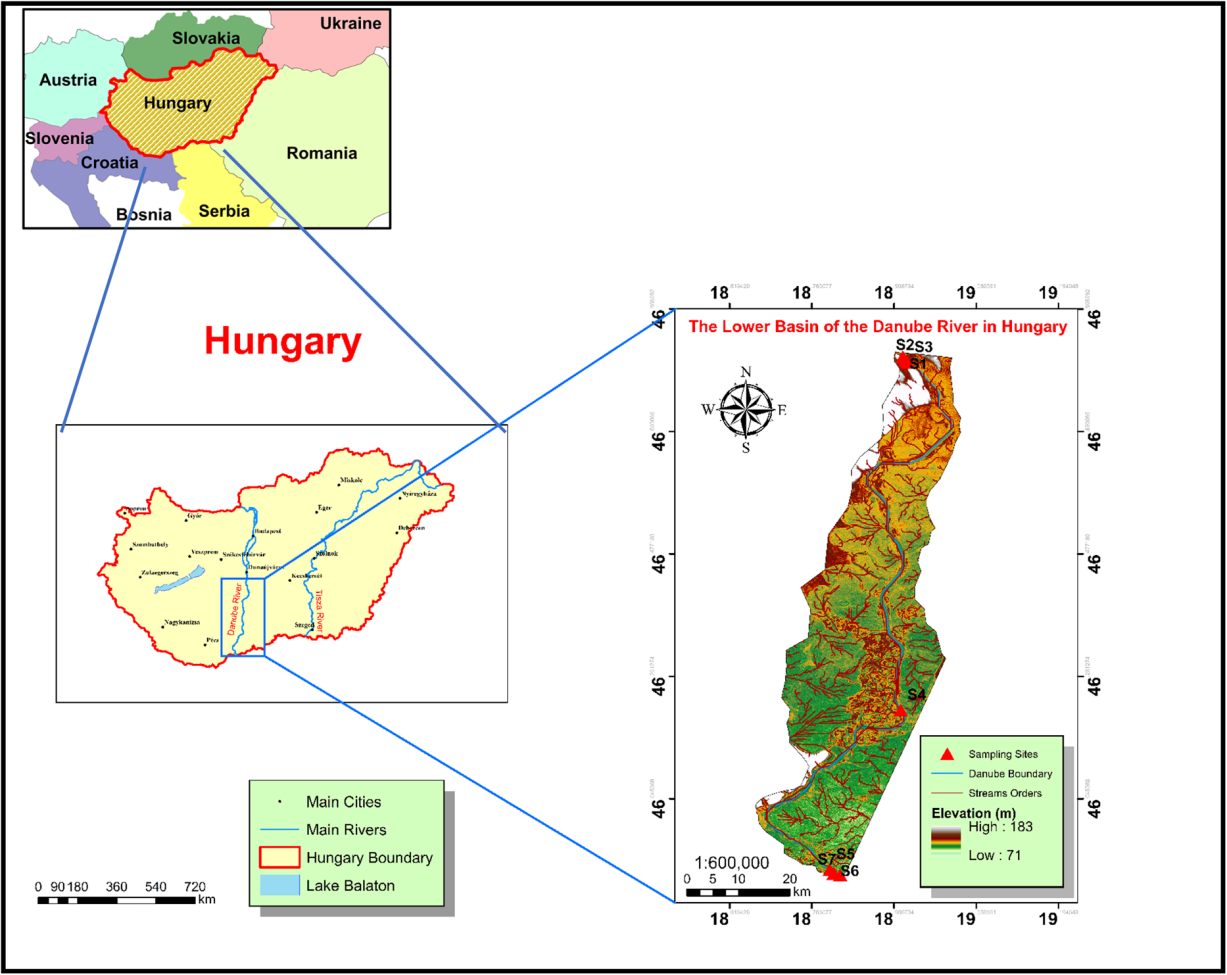
Study area and the distribution of sampling points in the Danube River

The study focused on the water quality of the Danube River in Hungary, specifically in Dunaföldvár, Baja, and Hercegszántó, over a period of seven years, from January 2013 to December 2019. Water samples were collected from seven different monitoring locations along the lower basin of the Danube River, which were located in the south of Hungary between 46° 10′ 54.4548′′ N and 18° 57′ 15.5016′′ E. These sites were identified as “S1, S2, S3” for the right, left, and middle streams of the Danube River in Dunaföldvár city, “S4” represents the left bank of the Danube River in Baja city; and “S5, S6, S7” for the right, left, and middle streams of the Danube River in Hercegszántó city, as illustrated on the map (Fig. 2). The samples were collected from these locations to cover different land use activities (industrial, agricultural, and urban areas) on the two sides of the river that could be sources of heavy metals. The total number of samples and locations were detected according to the regions that are characterized by intensive industrial, urban, and agricultural land.

The Danube River flows through several more countries after passing through these cities before ultimately emptying into the Black Sea. Hungary experiences a continental climate, characterized by hot summers with low humidity and frequent showers, as well as cold and snowy winters. Furthermore, Hungary also receives moderate precipitation throughout the year. Therefore, the regional weather can vary significantly from north to south. According to Hungary’s climatology from 1991 to 2020 (“Hungary—Climatology | Climate Change Knowledge Portal”), the average maximum temperature recorded during the period of July–August was 29 °C, and the average minimum temperature was recorded during the period of December–February (− 2.59 °C). The study area experiences heavy rainfall between May and July, with an average annual rainfall of 619 mm. The current study aims to: (i) identify potential sources of heavy metals by employing multivariate statistical techniques such as Spearman correlation analysis and heatmap cluster analysis along with the interpolation IDW technique; (ii) assess the potential non-carcinogenic and carcinogenic human health risks from heavy metals; and (iii) apply the Monte Carlo method to decrease uncertainty and predict the values of Hazard Quotients (HQ) for heavy metals.

### Samples preparation and heavy metal determination techniques

A total of 280 water samples were collected and placed in plastic bottles that had been pre-washed with acid. The samples were then filtered through a 0.45 mm cellulose nitrate membrane, acidified with diluted nitric acid, and stored in the fridge at − 4 °C before analysis. The concentrations of arsenic, chromium, copper, nickel, and lead were measured using electrothermal atomization atomic absorption spectrometry (ETA-AAS), while iron, manganese, and zinc were measured using flame atomic absorption spectrometry (FAAS). Consequently, Atomic Absorption Spectrometry was employed to analyze 500 mL of each sample. Subsequently, the acidified samples were divided into two 250 mL beakers, and their volume was reduced to 25 mL. An additional step was taken to analyze arsenic (As) samples, which were mixed with 0.5 mL of ascorbic acid, 0.5 mL of potassium iodide, and 3 mL of HCl for a 2-h period before being analyzed. This differed from the procedure for the other heavy metals in the study. Precision was ensured through triplicate analyses for each sample, resulting in relative standard deviations of ≤ 5%.

### Descriptive and multivariate statistical analysis

This study used Spearman correlation coefficient analysis (SC) to investigate the correlation between heavy metal concentrations. Heatmap cluster analysis (HCA) was applied to identify the relationships among the eight heavy metals existing in the study area and their potential sources. All data analysis and visualization were carried out using Python, while maps were prepared using QGIS software (version 3.16.4).

### Heavy metal pollution analysis index (HPI) and metal index (MI)

The heavy metal pollution Index (HPI) is considered the most effective method for assessing water contamination levels due to the presence of heavy metals (HMs) in water samples (Al-Hejuje1 et al. 2017). This index evaluates the suitability of water quality for human consumption based on metal contamination and considers the absolute nature of water in relation to heavy metals. The HPI is calculated using a weighted arithmetic mean approach, achieved by establishing a rating scale for each chosen parameter and assigning weights to each pollution parameter. The rating scale ranges from 0 to 1, and its determination is based on the significance of individual quality factors or can be established by comparing values relative to recommended standards for comparison (Shankar, 2019). The heavy metal pollution index (HPI) offers a mathematical assessment of water quality by considering the presence of heavy metals (HMs) in the water. The equation for calculating the HPI is provided below (Eqs. 1 and 2).

1 $${\text{HPI}} = \frac{{\mathop \sum \nolimits_{i = 1}^{n} W_{i} Q_{i} }}{{\mathop \sum \nolimits_{i = 1}^{n} W_{i} }}$$

where *Q*_*i*_ stands for the sub-index parameter; *n* is the number of parameters taken for analysis; *w*_*i*_ depicts the weight of each parameter, evaluated as 1/*S*_*i*_; *S*_*i*_ symbolize the standard value of each parameter; *Q*_*i*_ represents the sub-index of the boundary, determined by Eq. 2.

2 $$Q_{i} = \mathop \sum \limits_{i = 1}^{n} 100 \times \frac{{C_{i} }}{{S_{i} }}$$

The heavy metal pollution index (HPI) was calculated based on the concentration of eight HMs, namely chromium (Cr), copper (Cu), iron (Fe), manganese (Mn), nickel (Ni), lead (Pb), zinc (Zn), and one metalloid, arsenic (As) (due to its toxic characteristics).

A three-class modified scale is frequently used to accurately depict moderate levels of heavy metal pollution. This scale classifies heavy metal pollution as low (HPI < 15), medium (15 ≤ HPI ≤ 30), or high (HPI > 30) (Edet & Offiong, 2002; Qu et al., 2018).

On the other hand, the metal index (MI) of drinking water considers the cumulative potential impact of heavy metals (HMs) on human health and provides an evaluation of the overall quality of drinking water (Ojekunle et al., 2016). The MI assumes that the toxicity of HMs to organisms has a linear relationship with their concentration. HMs can cause a range of acute and chronic toxic effects on various body organs. The MI is calculated using a comprehensive assessment of the current situation. If the concentration of a metal exceeds its respective upper allowable limit (UAL) value, the quality of the drinking water will be degraded. The concept of MI was first introduced by Tamasai and Cini (Shankar, 2019) and can be expressed as follows (Eq. 3):

3 $$M_{i} = \mathop \sum \limits_{i = 1}^{i} \frac{{C_{{{\text{ave}}}} }}{{{\text{UAL}}_{i} }}$$

where *C*_ave_ signifies the average concentration of each studied HMs; UAL_*i*_ stands for the upper allowable limit of the ith metal in the sample. Metal index (MI) has 6 classes: very clean (MI < 0.3); clean (0.3 < MI < 1); partly affected (1 < MI < 2); moderately affected (2 < MI < 4); heavily affected (4 < MI < 6); and severally affected (MI > 6) (Withanachchi et al., 2018).

### The Potential ecological risk index of heavy metals

The potential ecological risk index (RI) for heavy metals, as introduced by Hakanson in 1980, is a technique employed to evaluate the potential risk associated with the presence of heavy metals in a specific ecosystem. This index takes into account factors such as the concentrations, types, sensitivity, toxicity, and background levels of the heavy metals, as noted by Xie et al. (2013). While it has been utilized across various scientific disciplines, in this study, it was employed to assess the ecological risks of heavy metals in river water. The formula is presented as follows (Eq. 4):

4 $${\text{RI}} = \sum E_{{\text{r}}}^{i} = T_{{\text{r}}}^{i} \times \left\{ {\frac{{C_{{{\text{ave}}}}^{i} }}{{C_{{{\text{bg}}}}^{i} }}} \right\}$$

where *E*_r_ indicates a substance’s potential ecological risk factor; *T*_r_ illustrates the given heavy metal toxic response factor (Table S1); ${C}_{\mathrm{ave}}^{\mathrm{i}}$ denotes the average concentration of each heavy metal in the sample; ${C}_{\mathrm{bg}}^{i}$ stands for the background values of each heavy metal (Table S1). RI is the overall contamination’s potential ecological risk. RI has four risk levels: low (below 30), moderate (30–60), considerable (60–120), and very high (over 120) (Yuan et al., 2015).

### Human health risk assessment of heavy metals

#### Non-carcinogenic human health risk method

The consumption of drinking water contaminated with toxic metals increases the potential for both non-carcinogenic and carcinogenic diseases in humans (Bineshpour et al., 2021; Qu et al., 2018). This study utilized methods outlined by the U.S. Environmental Protection Agency (Selvam et al., 2022) to assess the non-carcinogenic risks associated with As, Cr, Cu, Fe, Mn, Ni, Pb, and Zn (Selvam et al., 2022). The USEPA, (2004) established a health risk assessment approach to determine the non-cancer human health risks from heavy metal elements in groundwater and surface water through ingestion, inhalation, and skin contact. The primary risk stemmed from direct water consumption and absorption through the skin (Mukherjee et al., 2020; Qu et al., 2018; Saha et al., 2017; Saleem et al., 2019; Selvam et al., 2022). This method calculates the quantity of pollutants consumed by humans using the chronic daily intake (CDI) approach, which expresses the daily dose of pollutants in kilograms consumed through ingestion (CDI ingestion) and dermal absorption (CDI dermal) using Eqs. 5 and 6, respectively (Jehan et al., 2020; Tokatli & Ustaoğlu, 2020; USEPA, 2004).

5 $${\text{CDI}}_{{{\text{oral}}}} = \frac{{C_{{{\text{ave}}}} \times {\text{IR}} \times {\text{EF}}}}{{{\text{BW}} \times {\text{AT}}}} \times {\text{ED}}$$

6 $${\text{CDI}}_{{{\text{dermal}}}} = \frac{{C_{{{\text{ave}}}} \times {\text{ET}} \times {\text{EF}} \times K_{p} \times {\text{SA}} \times {\text{CF}}}}{{{\text{BW}} \times {\text{AT}}}} \times {\text{ED}}$$

where CDI represents the chronic daily intake (mg/kg/day); *C*_ave_ depicts the average concentration of each heavy metal (mg/L); IR stands for the intake rate (adult: 2.2 L day^−1^; child: 1.8 L day^−1^); EF denotes the exposure frequency (adult and child: 350 days/year); ED signifies the exposure duration (adult: 70 years; child: 6 years); ET represents the exposure time (adult: 0.58 h day^−1^; child: 1 h day^−1^); *K*_p_ is the permeability coefficient (cm/h) given in Table S1. SA depicts the skin area (adult: 18,000 ; child: 6600 cm^2^). BW is the body weight (adult: 70 kg; child: 15 kg); CF is the unit conversion factor (1 × 10^–3^ L cm^−3^); AT indicates the average time for carcinogenic risks (adult: 25,550 days; child: 2190 days) (Saleem et al., 2019; Selvam et al., 2022; Xu et al., 2020).

In the second step, we calculated the hazard quotient (HQ) by dividing the chronic daily intake (CDI) by the reference dose (RFD) for both oral and dermal exposure using Eqs. 7 and 8, respectivey (Imran et al., 2019; Mthembu et al., 2022; Saha & Paul, 2019).

7 $${\text{HQ}}_{{\text{dermal/oral}}} = \frac{{{\text{CDI}}_{{{\text{dermal}}}} {\text{/CDI}}_{{{\text{oral}}}} }}{{{\text{RfD}}_{{{\text{dermal}}}} {\text{/RfD}}_{{{\text{oral}}}} }}$$

8 $${\text{RfD}}_{{{\text{dermal}}}} = {\text{RfD}}_{{{\text{oral}}}} \times {\text{ABS}}$$

In the final step, the overall potential non-carcinogenic risks were assessed by calculating the hazard index (HI) using Eq. 9 (Jehan et al., 2020; Rupakheti et al., 2017).

9 $${\text{HI}} = {\text{HQ}}_{{{\text{oral}}}} + {\text{HQ}}_{{{\text{dermal}}}}$$

Toxic metals that have a hazard index (HI) or hazard quotient (HQ) of greater than 1 may pose negative impacts on human health, while those with a HI or HQ of less than 1 are considered to have no adverse effects (Selvam et al., 2022).

#### Carcinogenic human health risk method

Following Li and Zhang’s methodology (Li & Zhang, 2010), the degree of carcinogenic risk (CR) was ascertained using Eq. 10. The resulting value indicates the probability of developing cancer throughout one’s lifetime due to exposure to carcinogens. Normally, the acceptable or permissible range for such risks lies between 1 × 10^−6^ and 1 × 10^−4^ (Li & Zhang, 2010).

10 $${\text{CR}} = {\text{CDI}} \times {\text{ CSF}}$$

where CSF is the cancer slope factor (Table S1).

### Monte Carlo simulation approach and sensitivity analysis

**Fig. 3:**
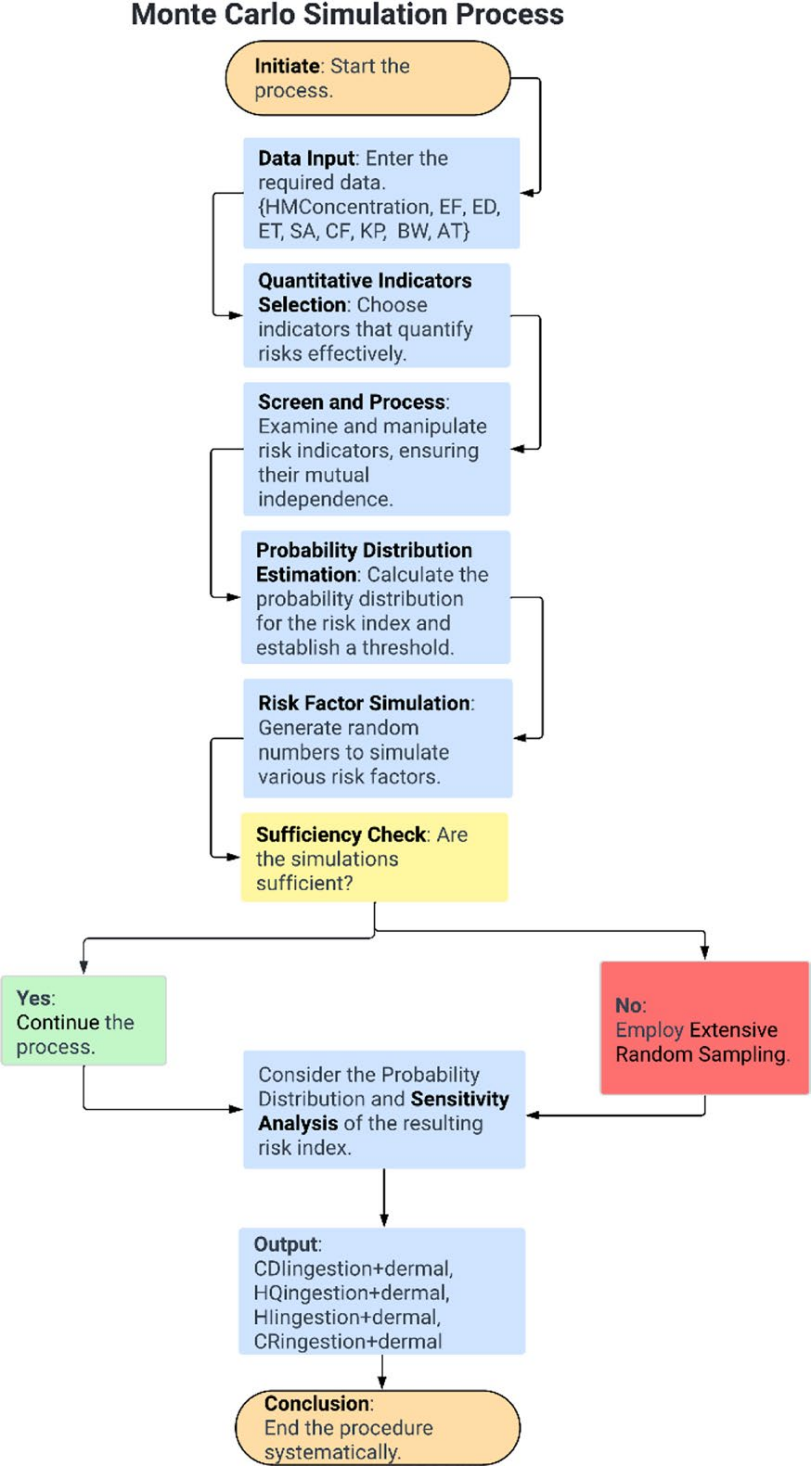
Operational sequence of a Monte Carlo simulation model

The primary objective of the Monte Carlo simulation in this study was to estimate the probability distributions for various factors, including heavy metal concentration, ingestion rate, exposure duration, exposure time, exposure frequency, average time, permeability coefficient, body weight, and skin-surface area (Fig. 3). This estimation was conducted to determine the uncertainty’s probability distribution associated with assessment metrics (Qu et al., 2018).

The utilization of Monte Carlo analysis in conjunction with the USEPA health risk assessment method enables the evaluation of uncertainty associated with human health risks for exposure to heavy metals by generating a probability distribution of CR values. Moreover, sensitivity analysis was performed to identify the most significant variables contributing to health risk, which is determined by the correlation coefficient between each parameter (e.g., concentration, intake rate, body weight, average time, exposure frequency, and exposure duration) and the risk value. A higher correlation coefficient signifies a greater contribution to the final health risk, and the sensitivity of each variable was expressed as a percentage (Qu et al., 2018). In the current study, the Monte Carlo method was applied to predict the HQ (oral and dermal) and CR (oral and dermal) for children and adults to decrease the uncertainty and increase the reliability of the non-carcinogenic and carcinogenic health risks for the heavy metals in the Danube River. The input parameters included the concentration of the heavy metals, and the factors mentioned previously (Eq. 5, 6, and 10). For the accuracy of the Monte Carlo simulation, the Python programming language ran 10,000 iterations, and the measured and predicted values of the HQ were similar, confirming the success of the model. While heavy metal concentration distributions were derived from available monitoring data between 2013 and 2019, the distributions of other parameters, including ingestion rate, exposure duration, body weight, and skin-surface area, were modeled as a normal distribution to reflect the true distribution of these parameters more accurately.

## Results and discussion

### Heavy metal concentrations in surface water

The present study investigated the distributions of eight heavy metals at seven representative sites, as illustrated in Table 1. Specifically, the concentrations of the following heavy metals—As, Cr, Cu, Fe, Mn, Ni, Pb, and Zn—were determined and averaged over two distinct periods: April–September and October–March. During April–September, the average concentrations of As, Cr, Cu, Fe, Mn, Ni, Pb, and Zn were 1.29, 1.43, 4.03, 520.63, 53.02, 2.70, 1.31, and 15.00 µg/L, respectively. The mean concentrations of these heavy metals were ranked in descending order: Fe > Mn > Zn > Cu > Ni > Cr > Pb > As. Conversely, during October–March, the average concentrations of As, Cr, Cu, Fe, Mn, Ni, Pb, and Zn were 1.44, 1.36, 3.69, 403.36, 39.71, 2.51, 1.27, and 15.59 µg/L, respectively. The mean concentrations of these determined heavy metals were ranked in descending order: Fe > Mn > Zn > Cu > Ni > As > Cr > Pb. Importantly, the mean concentrations of Fe and Mn exceeded the standard limits established by both the EU Directive and WHO in 2017.

**Table1:** The parameters for the computation of HQ, HI, RI and CR

HM	As	Cr	Cu	Fe	Mn	Ni	Pb	Zn	Ref
RfD Oral(mg/kg/day)	0.0003	0.003	0.04	0.7	0.024	0.02	0.0014	0.3	(Xu et al., 2020)
ABS	1	0.025	0.3	0.2	0.04	0.04	0.3	0.2	(Xu et al., 2020)
Rfd Dermal(mg/kg/day)	0.0003	0.000075	0.012	0.14	0.00096	0.0008	0.00042	0.06	(Xu et al., 2020)
CSFing mg/kg/day	1.5	0.5					0.5		(Xu et al., 2020)
CSFderm	50	500					500		(Xu et al., 2020)
*K*_p_	0.001	0.002	0.001	0.001	0.001	0.0002	0.0001	0.0006	(USEPA, 2004)
Background (µg/g)	10	30	30	15,000	500	20	20	100	(Woitke et al., 2003)
*T*_r_	10	2	5	1	1	5	5	1	(Hakanson, 1980)

### Geospatial modeling and analysis

**Fig. 4:**
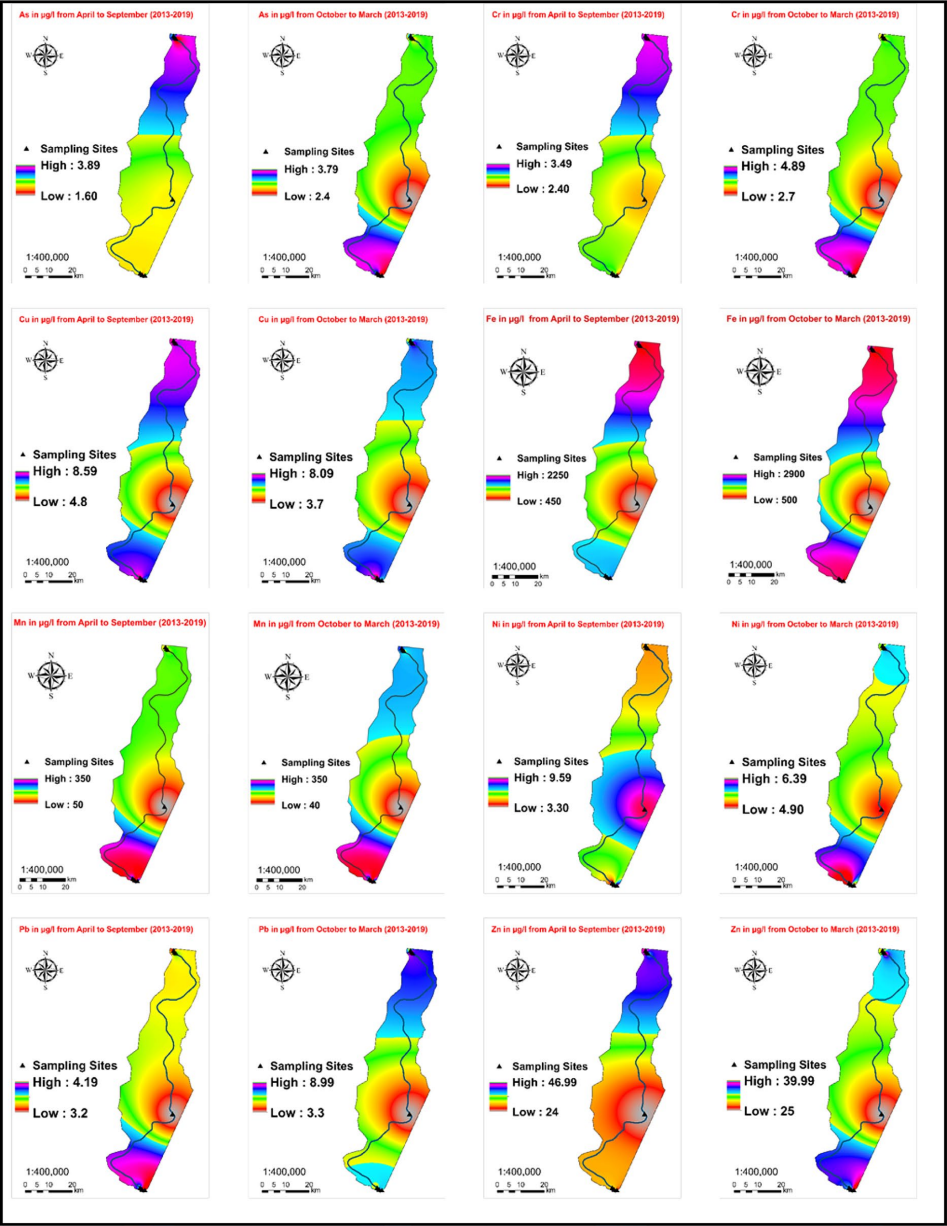
Concentrations and spatial distribution of the examined HMs in the study area (April–September & October–March, 2013–2019)

Figure 4 illustrates the spatial distribution of As, Cr, Cu, Fe, Mn, Ni, Pb, and Zn in the surface water of the lower basin of the Danube River in Hungary using a map generated by inverse distance weighting (IDW). The map depicts varying concentrations of heavy metals across different regions of the river. Notably, it showcases a significant disparity in the spatial distribution of the eight heavy metal elements. The majority of these heavy metals exhibited high concentrations both upstream and downstream of the study area. These areas are characterized by dense populations and industrial activities, resulting in the discharge of human and industrial waste into the river. Moreover, the excessive use of fertilizers in agriculture along the river contributes to the elevated levels of trace metals in the water.

#### Arsenic (As)

Elevated levels of arsenic in water have adverse impacts on both human health and the environment. Arsenic is a highly toxic substance and is associated with immune disorders, skin cancer, and reproductive dysfunction (Kacmaz, 2020; Selvam et al., 2022; Tokatli & Ustaoğlu, 2020). The concentration of arsenic in water was measured during two periods: April–September and October–March, with levels ranging from 0.9 to 3.90 µg/L and an average of 1.29 µg/L for the former, and from 0.1 to 3.79 µg/L with an average of 1.44 µg/L for the latter. These concentrations were found to be within the acceptable limits for drinking water as set by the EU and WHO, which is 10 µg/L, as shown in (Fig. 4; Table 2).

**Table 2:** The summary of the mean heavy metal concentrations in µg L^−1^ at the lower basin of the Danube River, Hungary

	Period	As	Cr	Cu	Fe	Mn	Ni	Pb	Zn
S1	April–Sep	1.21	1.45	4.29	**626.8**	**55.26**	2.55	1.22	16.45
	Oct–March	1.30	1.07	3.33	**405.5**	38.88	2.41	0.93	14.35
S2	April–Sep	1.27	1.3	4.17	**518.3**	48.26	2.64	1.61	14.78
	Oct–March	1.27	0.93	3.37	**336.1**	44.78	2.29	1.37	14.44
S3	April–Sep	1.34	1.42	4.4	**634.73**	**55.26**	2.64	1.42	17.66
	Oct–March	1.45	1.01	3.71	**452.9**	34.37	2.71	1.03	16.25
S4	April–Sep	1.35	1.8	3.48	170.67	40	3.1	1.14	12.93
	Oct–March	1.28	1.58	3.81	**208.3**	26	2.45	1.3	12.79
S5	April–Sep	1.37	1.11	3.74	**436.76**	43.42	2.87	1.2	11.98
	Oct–March	1.46	1.42	3.62	**381.2**	31.91	2.3	1.12	16.13
S6	April–Sep	1.20	1.49	4.11	**590.5**	**72.1**	2.86	1.45	14.38
	Oct–March	1.70	1.83	3.79	**511**	**65.71**	2.6	1.55	15.8
S7	April–Sep	1.31	1.44	4.03	**666.67**	**56.84**	2.27	1.14	16.8
	Oct–March	1.60	1.65	4.17	**528.5**	36.3	2.8	1.34	19.39
EU-Directive-2020/2184 (The European Parliament and the Council of the European Union 2020)		10	25.0	2000	200	50	20	10	_
WHO-2017 (WHO, 2017)		10	50.0	3000	300	50	70	10	1000

Bold values indicate that they have exceeded the given standards of EU and WHO

#### Chromium (Cr)

Despite reaching a peak concentration of 3.49 µg/L with an average of 1.43 µg/L during April–September, and increasing to 4.89 µg/L with an average of 1.36 µg/L during October–March, the levels of chromium (Cr) did not exceed the permissible limits established by the EU and WHO, which are 25 µg/L and 50 µg/L, respectively. The slight reduction in chromium concentration during April–September suggests a decrease in activities that contribute to chromium levels in the Danube River due to seasonal effects. This trend is positive for both the environment and public health.

#### Copper (Cu)

Copper is an essential nutrient for the human body (Samantara et al., 2017; Selvam et al., 2022). Our study revealed that copper concentrations were elevated during April–September, ranging from 2.6 to 8.59 µg/L with an average of 4.03 µg/L, and slightly decreased to a maximum of 8.09 µg/L with an average of 3.69 µg/L during October–March. However, the concentrations of copper remained within the acceptable limits set by the EU and WHO.

#### Iron (Fe)

The elevated iron (Fe) levels observed in surface water could potentially be attributed to various sources, including industrial waste discharge or non-point sources from rainwater runoff. Iron concentrations exceeding 300 µg/L have been reported to cause damage to laundry and plumbing components (Mollo et al., 2022; WHO, 2017). In the current study, iron concentrations varied from 10 to 2250 µg/L with an average of 520.6 µg/L during April–September and increased from 1 to 2900 µg/L with an average of 403.3 µg/L during October–March (Fig. 4; Table 2). The measured concentrations of iron (Fe) exceeded the acceptable limits set by the EU and WHO, which are 200 and 300 µg/L, respectively. Uzinger et al. (2015) reported that the Ajka red mud released during the 2010 Ajka alumina plant accident contained a high concentration of iron oxide, specifically Fe_2_O_3_, at around 40–45%. The significant iron concentration in the Danube River could likely be attributed to the deposition of iron after the Ajka accident in the Marcal and Raba rivers, which eventually flowed into the Danube River. This scenario appears to be the most plausible cause contributing to the elevated iron levels in the study area.

#### Manganese (Mn)

Although manganese is an essential element, excessive exposure to it can have detrimental effects on human health (Obasi & Akudinobi, 2020). In this study, manganese concentrations ranged from 5 to 350 µg/L with an average of 53.02 µg/L during April–September, and from 5 to 350 µg/L with an average of 39.7 µg/L during October–March. However, the levels of manganese in some sites exceeded the allowable limits set by both the EU and WHO for both periods, which are 50 µg/L (Fig. 4; Table 2).

#### Nickel (Ni)

As indicated in Table 2 and Fig. 4, nickel concentrations ranged from 1.6 to 9.59 µg/L with a mean of 2.70 µg/L during April–September, and from 0.7 to 6.39 µg/L with a mean of 2.51 µg/L during October–March. These values were found to be within the permissible limits set by the EU and WHO, which are below 20 µg/L and 70 µg/L, respectively. It is noteworthy that nickel is considered a carcinogenic metal by the International Agency for Research on Cancer (IARC) (Mollo et al., 2022; WHO, 2017).

#### Lead (Pb)

Lead levels ranged from 0.2 to 4.19 µg/L with an average of 1.31 µg/L during April–September, and from 0.3 to 8.99 µg/L with an average of 1.27 µg/L during October–March. However, all samples were found to fall within the permissible range for drinking water (Fig. 4; Table 2). The decrease in lead concentration might be attributed to a reduction in the release of industrial effluents and petroleum-related transportation into the river.

#### Zinc (Zn)

Zinc plays a crucial role in human metabolism and immune function (Karunanidhi et al., 2021; Samantara et al., 2017; Selvam et al., 2022). In this study, zinc concentrations ranged from 2.1 to 46.99 µg/L with an average of 15.0 µg/L during April–September, and from 7.2 to 40 µg/L with an average of 15.59 µg/L during October–March. These levels did not exceed the permissible limits set by the EU and WHO, indicating their suitability for drinking purposes (Fig. 4; Table 2).

**Fig. 5:**
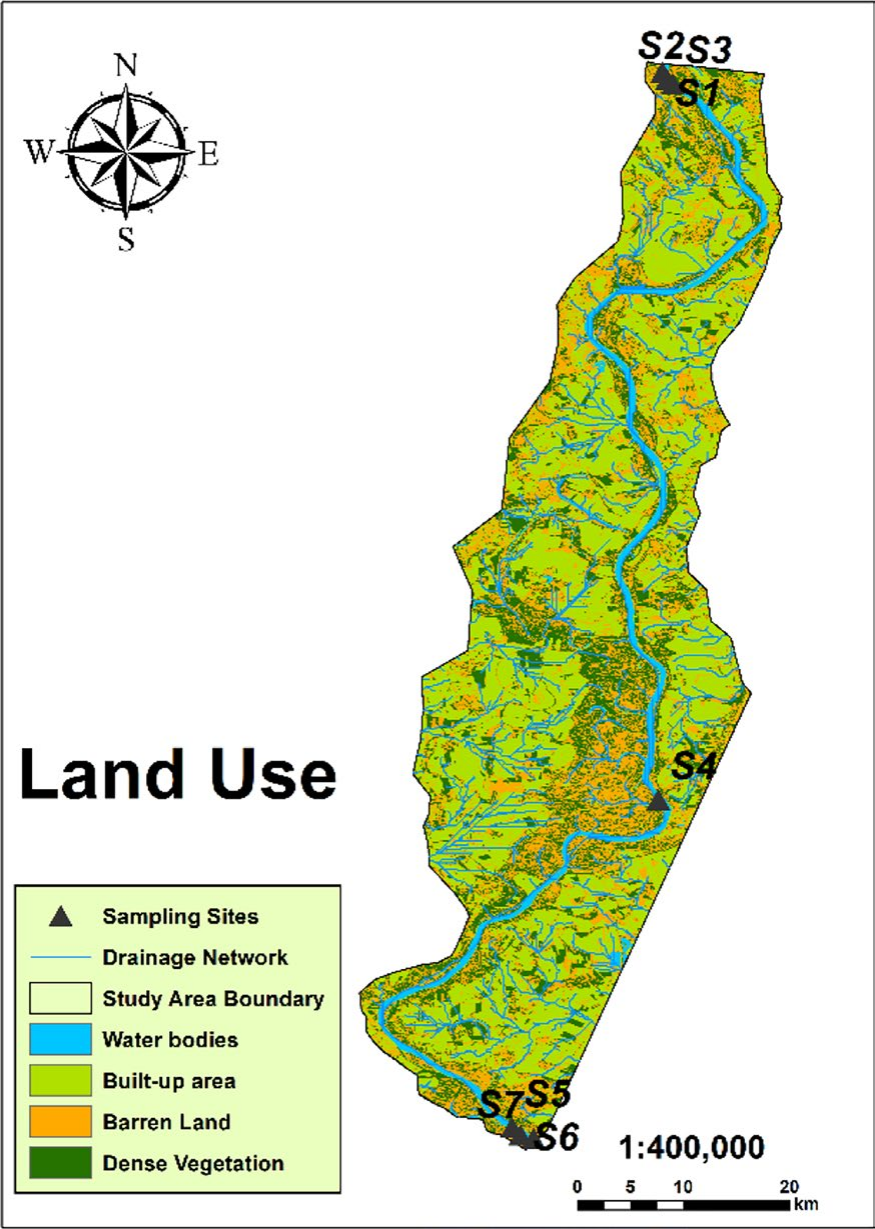
Land use map of the study area

The observed fluctuations in heavy metal concentrations in the water samples during both aforementioned periods can be attributed to contributions from both natural processes and human activities (Sharma et al., 2019). Natural factors such as variations in weather patterns, geological characteristics, and soil composition can influence the levels of heavy metals in surface water. Conversely, anthropogenic actions, including industrial and agricultural practices, can introduce heavy metals into surface water. Hence, ongoing monitoring and assessment of heavy metal sources and levels in surface water are crucial for comprehending potential risks to human health and the environment. Table S2 presents the concentrations of certain heavy metals (loids) in the lower basin of the Danube River across Serbia and Romania during different seasons from 2007 to 2012, alongside the concentration of certain heavy metals in various global rivers. The first three positions correspond to the point where the river is initially crossed downstream from the Ajka accident site, with the incident occurring upstream. In these locations (S1, S2, and S3), iron (Fe) and manganese (Mn) concentrations significantly surpass the standards set by both the World Health Organization (WHO) and the European Union (EU). The elevated levels of Fe and Mn in these sites can be attributed to the Ajka accident, which released substantial amounts of these metals into the river. As the polluted water progresses downstream, it gradually reaches these initial positions, leading to the observed high concentrations. Upon reaching the mid-position (S4), Fe and Mn levels decrease, suggesting dilution as water from additional sources mixes with the contaminated water, leading to reduced concentrations. However, as the river advances toward the final positions (S5, S6, and S7), Fe and Mn concentrations start to rise again. This escalation indicates that sources beyond the Ajka accident contribute to the elevated levels of these elements. The presence of agricultural and urban areas along the riverbanks in the study area implies that these sources could be responsible for the heightened Fe and Mn concentrations observed in the last three positions, as depicted in Fig. 5.

### Heavy metal pollution index (HPI) and metal index (MI)

**Fig. 6:**
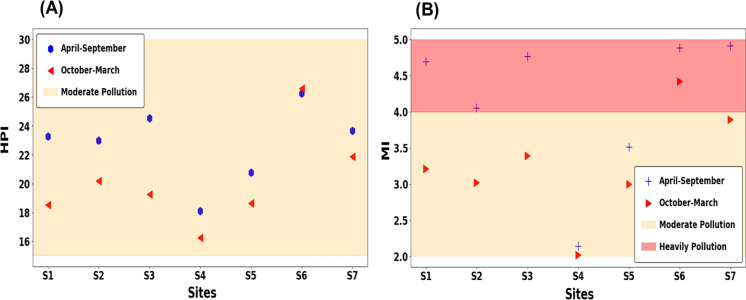
Heavy metal pollution index (HPI) (**A**) and metal index (MI) (**B**)

The heavy metal pollution index (HPI) model serves as a valuable tool for evaluating the overall pollution level in surface water. This model enables the assessment of heavy metal impacts on water quality and aids in the monitoring and management of potential health risks associated with heavy metal exposure (Rahman et al., 2022). The highest HPI values for the studied heavy metal elements in the lower basin of the Danube River were observed during the October–March period. The mean HPI value for April–September was recorded at 21.91, ranging from 17.63 to 25.17, while the mean HPI value for October–March was 19.42, ranging from 15.82 to 25.59. Based on the findings and referring to Edet and Offiong (2002) (Edet & Offiong, 2002), nearly 100% of the samples taken in both periods fell within the moderate pollution range of the HPI (15–30). Notably, the surface water was found to be mostly free from the studied heavy metal contamination, except for iron (Fe) and manganese (Mn). However, the presence of significant quantities of Fe and Mn at all sites contributed to elevated HPI values at those sampling locations. (Fig. 6A).

To comprehend the impact of heavy metals on water quality, the MI (Metal Index) method was employed alongside the HPI index to assess the level of heavy metal contamination in water, utilizing the upper allowable limit values outlined in the EU-Directive Framework guideline. The average MI values for April–September and October–March were determined to be 4.13 and 3.28, respectively. These values indicate that the water samples exhibited severe contamination during April–September and moderate contamination during October–March. The extent of contamination, relative to the upper limit values set by the EU-Directive Framework guideline, suggests a significant impact and contamination of heavy metals in the water samples. This underscores the need for urgent monitoring and enhancement of water quality in the lower basin of the Danube River in Hungary. (Fig. 6B).

### Spearman correlation and heatmap cluster analysis

**Fig. 7:**
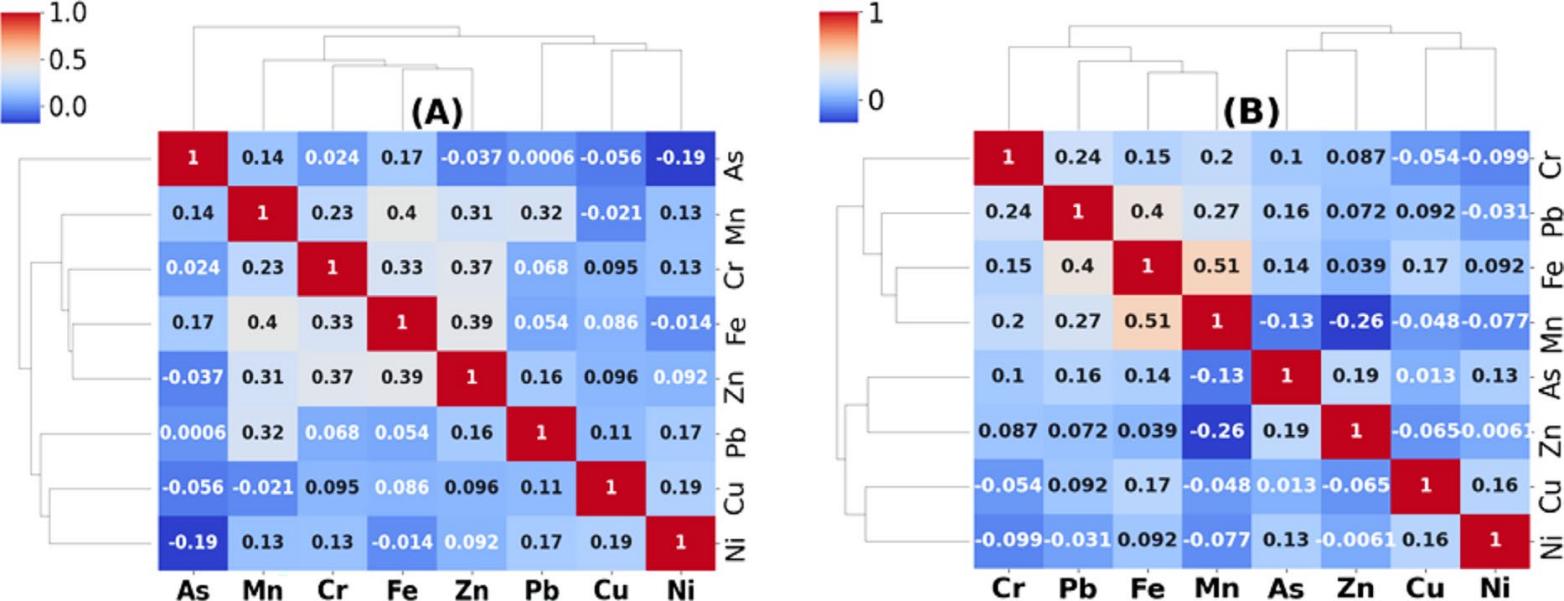
Heatmap and Spearman correlation for the studied heavy metal: **A** during April–September; **B** during October–March

The normality of the data was assessed using the Kolmogorov–Smirnov test prior to conducting the statistical analysis. The relationships among the various heavy metal parameters in the Danube surface water can provide insights into their sources and movements. For non-normally distributed parameters, the nonparametric Spearman correlation matrix was utilized, as depicted in Fig. 7. In the period of April–September (Fig. 7A), with a significance level of *p* < 0.01, several positive correlations were observed. Specifically, there was a positive correlation between chromium (Cr) and iron (Fe) (*r* = 0.33), between Cr and manganese (Mn) (*r* = 0.23), and between Cr and zinc (Zn) (*r* = 0.37). Similarly, positive correlations were found between Fe and Mn (*r* = 0.40), Fe and Zn (*r* = 0.39), as well as between Mn and lead (Pb) (*r* = 0.32), and between Mn and Zn (*r* = 0.31). Additionally, at a significance level of *p* < 0.05, there were positive correlations between arsenic (As) and Fe (*r* = 0.17), between copper (Cu) and nickel (Ni) (*r* = 0.19), and between Ni and Pb (*r* = 0.17). The significant positive correlations between Cr, Fe, Mn, Zn, and Pb at a significance level of *p* < 0.01 imply a common source for these elements. Conversely, the positive correlations between Ni, Cu, As, and Zn at a significance level of *p* < 0.05 suggest diverse origins from which these elements may arise (Table 3).

**Table 3:** The reported toxic metals in the lower Danube River and other global rivers in µg L^−1^

Sampling area/Country	Survey period	Heavy metals								Ref.
		Cr	Pb		Cu	Zn	Fe	Ni	As	
Holbina-Dunavat/RO	2007	_	42.6		–	334	–	86	–	(Burada et al., 2014)
Sontea-Fortuna/RO		_	21		–	144	–	104	–	
Matita-Merhei/RO		_	24		–	231	–	68	–	
Somova-Parches/RO		_	9.3		–	176	–	64	–	
Holbina-Dunavat/RO	2008	_	48		–	249.6	–	38	–	
Sontea-Fortuna/RO		_	34.7		–	124	–	32	–	
Matita-Merhei/RO		_	36		–	165	–	27	–	
Somova-Parches/RO		_	8		–	168	–	48	–	
Holbina-Dunavat/RO	2009	_	32		–	311	–	41	–	
Sontea-Fortuna/RO		_	28		–	178.9	–	88	–	
Matita-Merhei/RO		_	29		–	187	–	39	–	
Somova-Parches/RO		_	6.8		–	128	–	36	–	
Holbina-Dunavat/RO	2010	_	36		–	312	–	98	–	
Sontea-Fortuna/RO		_	11		–	197	–	92	–	
Matita-Merhei/RO		_	23		–	182.2	–	89	–	
Somova-Parches/RO		_	7		–	126	–	49	–	
Holbina-Dunavat/RO	2011	_	39		–	209	–	55	–	
Sontea-Fortuna/RO		_	17		–	172	–	41	–	
Matita-Merhei/RO		_	32		–	161	–	85	–	
Somova-Parches/RO		_	6.9		–	151	–	48	–	
Somova Lake/RO	2007–2012 Spring	_	7.7–11		–	161–209.8	–	49–78.9	–	(Burada et al.,)
Rotundu Lake/RO		_	6.8–8.9		–	124–181	–	40–59	–	
Somova Lake/RO	2007–2012 Summer	_	6.1–7.5		–	138–188.7	–	41–59	–	
Rotundu Lake/RO		_	6.2–7.2		–	100–164	–	27.9–46.6	–	
Somova Lake/RO	2007–2012 Autumn	_	6.8–8.9		–	147–179.6	–	43–71	–	
Rotundu Lake/RO		_	6.2–7.8		–	117.7–179.3	–	36–52.8	–	
Babadag Lake/RO	2008	June	_	1.6	–	–	–	2	2	(Rusu et al., 2014)
		July	_	1.5	–	–	–	2.1	2.1	
		September	_	1.7	–	–	–	2.3	1.9	
	2009	June	_	1.5	–	1.2	–	2.1	2.2	
		July	_	1.5	–	1.3	–	2.1	2.4	
		September	_	1.5	–	1.1	–	2.3	2.3	
	2010	June	_	1.5	–	1.2	–	2	2.9	
		July	_	1.5	–	1.3	–	2.1	2.8	
		September	_	1.5	–	1.1	–	2.3	2.7	
Galati/RO	2010	August	_	21.4 ± 1.6	112.3 ± 3.8	47.1 ± 2.0	–	–	–	(Ioniţă et al., 2014)
Tulcea/RO			_	14.3 ± 2.5	93.5 ± 2.5	32.5 ± 1.6	–	–	–	
Novi Sad/SE	2011	June	_	0.6	6	20	260	1.4	1	(Milošković et al., 2016)
Zemun/SE	2013	October	_	0.5	6	20	210	3	0.6	
Radujevac/SE			_	0.5	6	9	80	0.9	2	
Coronini,Caras-Severin/RO	2010 December–2012 July	_	_		1.46 ± 0.59	0.78 ± 0.86	–	–	–	(Matache et al., 2013)
Bazias/RO		_	_		1.89 ± 0.04	1.8 ± 3.49	–	–	–	
Divici/RO		–	–		3.17 ± 1.86	1.35 ± 1.89	–	–	–	
Batajnica, Belgrade/SE	2012	Autumn	_	ND	4 ± 1	32 ± 2	330			(Raša et al., 2016)
Xiangxi River, China	2021		_	10.59 ± 7.33	3.21 ± 0.83	2.4 ± 2.61		6.2 ± 1.66	0.65 ± 0.46	(Wang et al., 2015)

During the October–March period (Fig. 7B), positive correlations were observed at a significance level of *p* < 0.01. Specifically, lead (Pb) and chromium (Cr) were positively correlated (*r* = 0.24), as were Pb and iron (Fe) (*r* = 0.40), Pb and manganese (Mn) (*r* = 0.272), and Fe and Mn (*r* = 0.515). Furthermore, at a significance level of *p* < 0.05, zinc (Zn) was positively correlated with arsenic (As) (*r* = 0.19), Mn with Cr (*r* = 0.199), and Fe with copper (Cu) (*r* = 0.17). The significant positive correlations between Cr, Fe, Mn, and Pb at a significance level of *p* < 0.01 during October–March indicate a possible shared source for these elements. On the other hand, the positive correlations between As, Zn, Cu, and Ni at a significance level of *p* < 0.05 suggest diverse origins for these elements. The heatmap cluster tree is a valuable visualization tool for identifying clusters of variables with similar correlation patterns in a correlation matrix. The results of the heatmap cluster tree analysis in this study were consistent with the findings from the Spearman correlation matrix, which was used to analyze non-normally distributed parameters. The positive correlations between the studied heavy metals, such as Cr, Fe, Mn, Zn, and Pb, observed in the Spearman correlation matrix were also evident in the heatmap cluster tree. Similarly, the diverse origins of heavy metals such as As, Zn, Cu, and Ni identified in the Spearman correlation matrix were also apparent in the heatmap cluster tree. These findings underscore the utility of the heatmap cluster tree as a complementary tool to the correlation analysis matrix, particularly for non-normally distributed data, in revealing correlation patterns among variables. In terms of heavy metal movement, storm runoff can transport heavy metals from various sources (industrial, transportation, and human activities) into rivers (Xiong et al., 2021). As a result, heavy metals can be introduced to river water from reservoir banks and hydrofluctuation areas. When favorable environmental conditions (such as redox potential and pH) are present, heavy metals in sediments can be released into the water, leading to heavy metal pollution (Gao et al., 2018). Thus, it is important to implement technical solutions to mitigate heavy metal pollution in bank soil, such as ecosystem conservation and restoration through afforestation, reduction of peripheral businesses, and conversion of cropland to forest (Xiong et al., 2021). Manganese, being widely distributed in the Earth’s crust and often associated with iron, suggests a connection between Mn and Fe. The cluster analysis grouped the heavy metals into three main clusters:

Cluster 1 (Fe, Cr, Mn, Zn): This group likely indicates metals that might share similar sources or behaviors in the river. These metals could potentially have a common origin, possibly linked to industrial processes, urban runoff, or agricultural activities. Cluster 2 (Pb, Ni, Cu): The second cluster includes metals that exhibit a different pattern from the first group. This suggests that they might have separate sources or behave differently in the river system. These sources could be related to industrial discharges, traffic pollution, or other anthropogenic activities. Cluster 3 (As): Arsenic (As) forms its own cluster, indicating that it may have distinct sources and behaviors compared to the other metals. Arsenic contamination could be influenced by various factors such as geological sources, agricultural practices, or industrial discharges. Strong Positive Correlations: The correlation values (0.4, 0.32, 0.31) between Mn and Fe, Mn and Zn, and Mn and Pb indicate moderate to strong positive relationships. This could imply that these metals share common sources or that their behavior in the river is interconnected. For example, the correlation between Mn and Fe might suggest a connection between iron-rich sediments and manganese. Moderate Positive Correlations: The correlations between Cr and Fe (0.33) and Cr and Zn (0.37) suggest moderate positive relationships. Similar to the above point, this could indicate co-occurrence due to shared sources or similar environmental behaviors. Low Correlations: The fact that Ni and As have correlations below 0.1 with all other metals suggests that their presence might be influenced by different factors or sources, and they may not exhibit strong interdependencies with the other metals in the dataset. Industrial Sources: Metals in the same clusters, like Cluster 1 (Fe, Cr, Mn, Zn), might suggest industrial inputs as a potential source, possibly from industrial discharges or effluents. Urban and Agricultural Sources: Considering the Danube River’s location near urban and agricultural areas, metals like Cu, Pb, and Zn might come from urban runoff, traffic emissions, and agricultural practices. Geological Sources: Elements like As might have geological sources, possibly arising from the natural composition of the soil and rock in the region. These findings could be valuable for understanding pollution sources, planning environmental management strategies, and implementing measures to mitigate heavy metal contamination in the Danube River.

### The potential ecological risk index (RI)

**Fig. 8:**
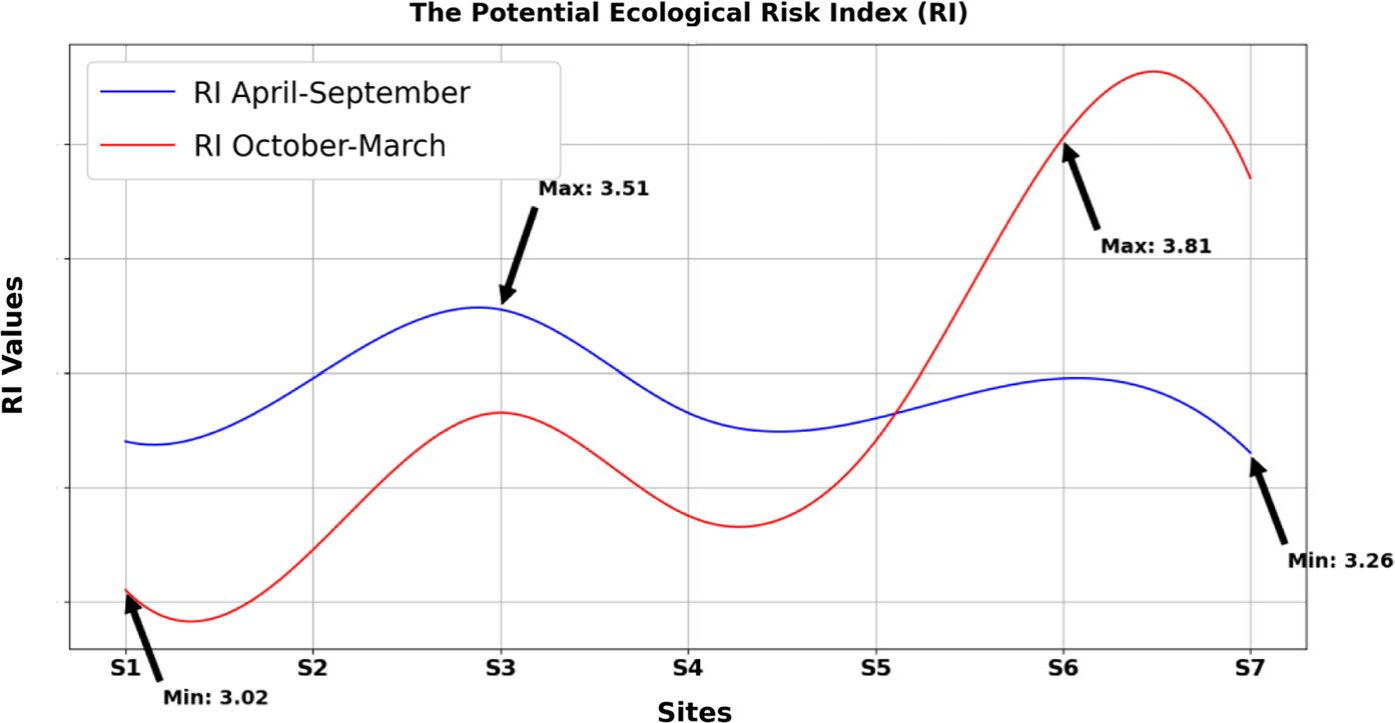
Ecological risk index (RI)

The potential ecological hazard index, developed by Hakanson in 1980, is a widely recognized method used for evaluating the extent of heavy metal pollution and its potential impact on both sediment and water environments. This index considers a range of factors, including the concentration of heavy metals, their ecological and toxicological effects, as well as their broader environmental implications. By integrating these factors, the index provides a comprehensive assessment of the ecological risks associated with heavy metal pollution in sediment and water systems. In this study, the potential ecological risk index (RI) for heavy metals was examined, as depicted in Fig. 8. The calculated average RI values for samples collected during the periods of April–September and October–March were 3.35 and 3.34, respectively. These findings suggest that the heavy metals investigated in the lower basin of the Danube River in Hungary pose a relatively low ecological risk (RI < 30). Figure 8 illustrates the potential ecological risk index (RI) in relation to the sampling locations within the study area. This visualization helps demonstrate the connection between the degree of pollution and the associated ecological risk. The study area is characterized by the presence of significant agricultural and residential zones located near the river and its inflow streams. These factors could contribute to the accumulation of higher concentrations of heavy metals in sediment, which can subsequently make their way into the Danube River. Therefore, the assessment of potential ecological risks related to heavy metal contamination in aquatic ecosystems is of paramount importance. This assessment informs the development of effective management strategies and policies aimed at mitigating the potential hazards posed by these trace pollutants.

### Health risk assessment

The non-carcinogenic and carcinogenic risk hazard indices (HI) were assessed by calculating the hazard quotients (HQ) for both ingestion and dermal absorption pathways. The outcomes reveal the combined potential health risks for humans resulting from exposure to different heavy metals during both the April–September and October–March time frames for both children and adults.

#### Non-carcinogenic health risk

**Fig. 9:**
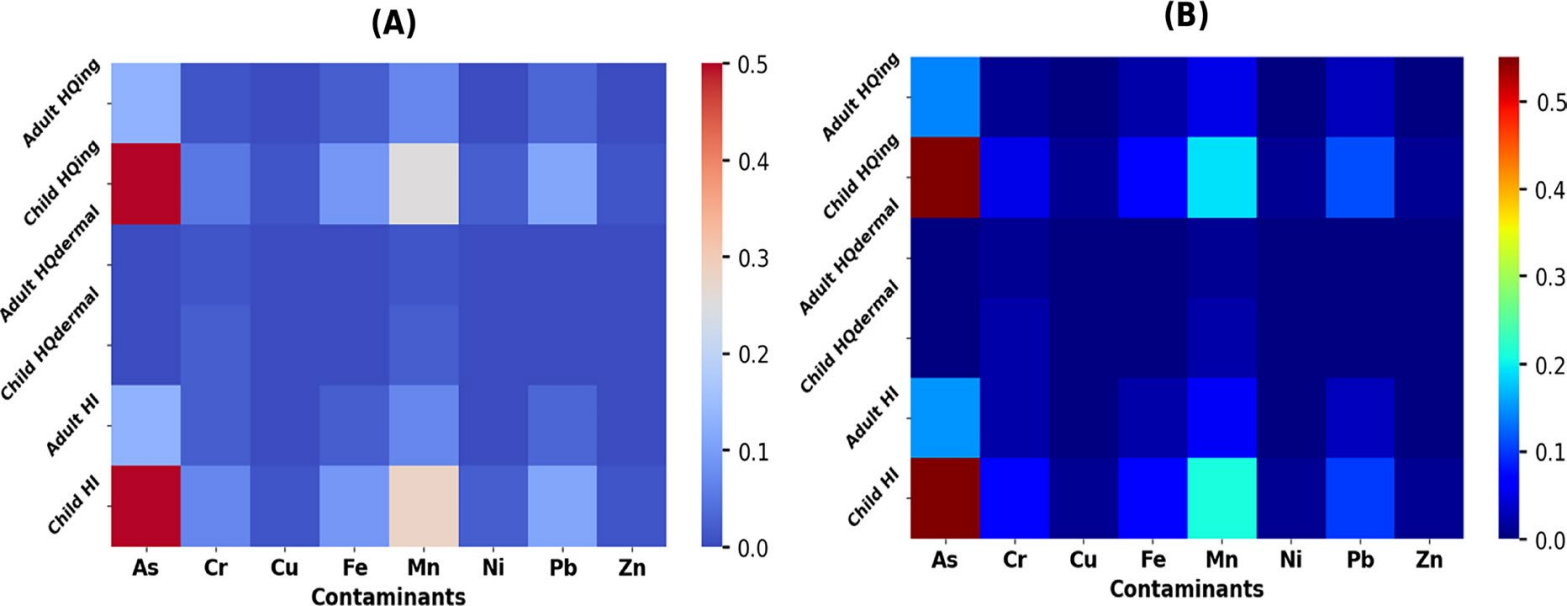
Hazard quotient and Hazard index for oral and dermal pathways: **A** during April–September; **B** during October–March

The toxic elements arsenic (As), chromium (Cr), copper (Cu), iron (Fe), manganese (Mn), nickel (Ni), lead (Pb), and zinc (Zn) were evaluated to determine the non-carcinogenic risk in both children and adults (Fig. 9). During the April–September period for adults, the HQ ingestion ranged from 1.2E−1 to 1.4E−1, 1.1E−2 to 1.8E−2, 2.6E−3 to 3.3E−3, 7.3E−3 to 2.9E−2, 5.0E−2 to 9.1E−2, 3.4E−3 to 4.7E−3, 2.5E−2 to 3.5E−2, and 1.2E−3 to 1.8E−3 for As, Cr, Cu, Fe, Mn, Ni, Pb, and Zn, respectively. The HQ ingestion for children for the same period ranged from 4.6E−1 to 5.3E−1, 4.3E−2 to 6.9E−2, 1.0E−2 to 1.3E−2, 2.8E−2 to 1.1E−1, 1.9E−1 to 3.5E−1, 1.3E−2 to 1.8E−2, 9.4E−2 to 1.3E−1, and 4.6E−3 to 6.8E−3 for As, Cr, Cu, Fe, Mn, Ni, Pb, and Zn, respectively (Fig. 9A). Conversely, during the October–March period, the HQ ingestion for adults ranged from 1.3E−1 to 1.7E−1, 9.3E−3 to 1.8E−2, 2.5E−3 to 3.1E−3, 9.0E−3 to 2.3E−2, 3.3E−2 to 8.3E−2, 3.5E−3 to 4.2E−3, 2.1E−2 to 3.3E−2, and 1.3E−3 to 1.9E−3 for As, Cr, Cu, Fe, Mn, Ni, Pb, and Zn, respectively. The HQ ingestion for children for the same period ranged from 4.9E−1 to 6.5E−1, 3.6E−2 to 7.0E−2, 9.6E−3 to 1.2E−2, 3.4E−2 to 8.7E−2, and 1.2E−1 to 3. 2E−1, 1.3E−2 to 1.6E−2, 8.1E−2 to 1.3E−1, and 4.9E−3 to 7.4E−3 for As, Cr, Cu, Fe, Mn, Ni, Pb, and Zn, respectively. (Fig. 9B).

Based on HQ oral values, it seems that the human health risks associated with exposure to As, Cr, Cu, Fe, Mn, Ni, Pb and Zn through ingestion are generally higher for child than for adult during April–September period. It is worth noting that though HQ oral values are within the permissible limit under 1, these values are specific to the location and period studied and that the actual human health risks may vary depending on various factors such as exposure duration and frequency, individual susceptibility, and environmental conditions.

Nonetheless, during April–September, the HQ dermal values for adults were in the range of 6E−4 to 7E−4, 4.2E−3 to 6.9E−3, 1E−5 to 4E−4, 2E−4 to 7E−4, 6E−3 to 1.07E−2, 1E−4 to 3E−4, 0 to 1E−5, and 1E−5 to 3E−5 for As, Cr, Cu, Fe, Mn, Ni, Pb, and Zn, respectively. Moreover, for children during the same period, the HQ dermal values were in the range of 1.7E−3 to 1.9E−3, 1.25E−2 to 2.03E−2, 1E−4 to 2E−4, 5E−4 to 2E−3, 1.76E−2 to 3.17E−2, 2E−4 to 3E−4, 1E−4 to 2E−4, and 1E−4 to 1E−4 for As, Cr, Cu, Fe, Mn, Ni, Pb, and Zn, respectively (Fig. 9A). In contrast, during October–March, the HQ dermal values for adults were in the range of 6E−4 to 8E−4, 3.5E−3 to 7E−3, 2E−5 to 4E−5, 2E−4 to 5E−4, 3.9E−3 to 9.8E−3, 1E−4 to 2E−4, 1E−5, 2E−4, and 1E−5 to 2E−5 for As, Cr, Cu, Fe, Mn, Ni, Pb, and Zn, respectively. For children during the same period, the HQ dermal values were in the range of 1.8E−3 to 2.4E−3, 1.05E−2 to 2.06E−2, 1E−4 to 1E−4, 6E−4 to 1.6E−3, 1.14E−2 to 2.89E−2, 2E−4 to 3E−4, 1E−4 to 2E−4, and 1E−4 to 1E−4 for As, Cr, Cu, Fe, Mn, Ni, Pb, and Zn, respectively. (Fig. 9B). Based on HQ oral and dermal values, it seems that the human health risks associated with exposure to As, Cr, Cu, Fe, Mn, Ni, Pb and Zn through dermal exposure are generally higher for child than for adult during October–March. It is worth noting that though HQ dermal values are within the permissible limit under 1, these values are specific to the location and period studied and that the actual human health risks may vary depending on various factors such as exposure duration and frequency, individual susceptibility, and environmental conditions. The lower basin of the Danube River in Hungary used the hazard index (HI) to show the overall potential health risk that heavy metals pose. This was determined by adding up the hazard quotients (HQs) associated with all conceivable exposure routes, including ingestion and dermal pathways. During April–September, the hazard index (HI) values for adults were 1.3049E−1, 1.9819E−2, 3.0854E−3, 2.2947E−2, 7.4476E−2, 4.1716E−3, 2.8275E−2, and 1.5280E−3 for As, Cr, Cu, Fe, Mn, Ni, Pb, and Zn, respectively. Moreover, for children during the same period, the HI values were 4.9771E−1, 7.0938E−2, 1.1739E−2, 8.7153E−2, 2.7751E−1, 1.5844E−2, 1.0792E−1, and 5.8156E−3 for As, Cr, Cu, Fe, Mn, Ni, Pb, and Zn, respectively (Fig. 9A). On the other hand, during October–March, the HI values for adults were 1.4506E−1, 1.8789E−2, 2.8208E−3, 1.7778E−2, 5.5776E−2, 3.8697E−3, 2.7320E−2, and 1.5887E−3 for As, Cr, Cu, Fe, Mn, Ni, Pb, and Zn, respectively. For children during the same period, the HI values were 5.5325E−1, 6.7253E−2, 1.0732E−2, 6.7521E−2, 2.0783E−1, 1.4697E−2, 1.0428E−1, and 6.0466E−3 for As, Cr, Cu, Fe, Mn, Ni, Pb, and Zn, respectively. (Fig. 9B).

According to the HI values, it can be concluded that the HI for both adult and child were within safe levels during October–March. None of the HI values exceeded 1. However, it is still essential to monitor the levels of these metals in the Danube river and their potential health effects, especially for vulnerable populations such as children where the Danube river is one of the main water resources used for drinking and irrigation in Hungary.

### Monte Carlo simulation approach and sensitivity analysis

The Monte Carlo simulation was applied to predict the values of HQ (oral and dermal) of As, Cr, Cu, Fe, Mn, Ni, Pb, and Zn as well as CR (oral and dermal) of As, Cr, and Pb for both adults and children.

#### Non-carcinogenic risk

**Fig. 10:**
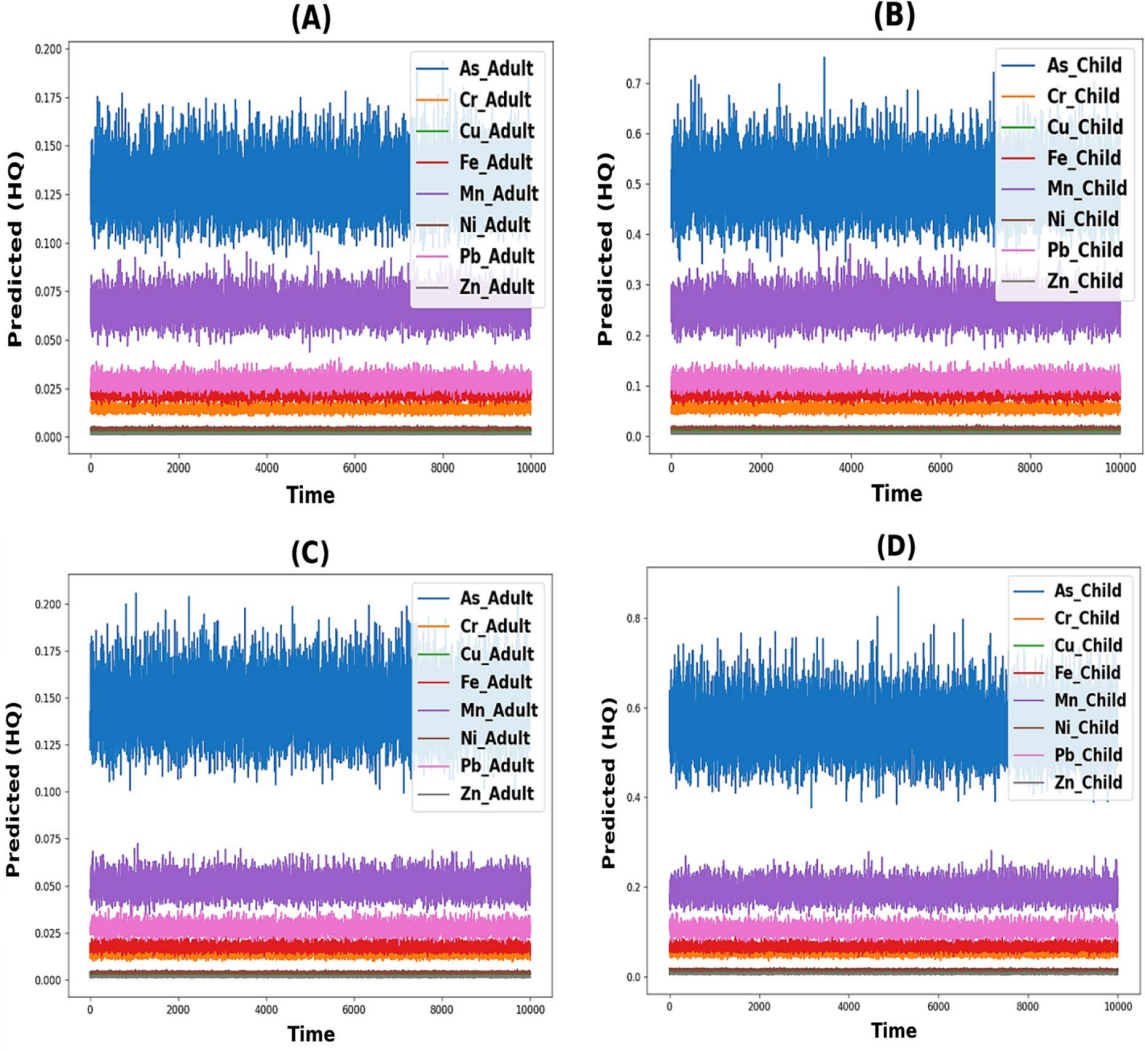
Predicted oral hazard quotient **A** Adults oral during April–September; **B** Children oral during April–September; **C** Adults oral during October–March & **D** Children oral during October–March

**Fig. 11:**
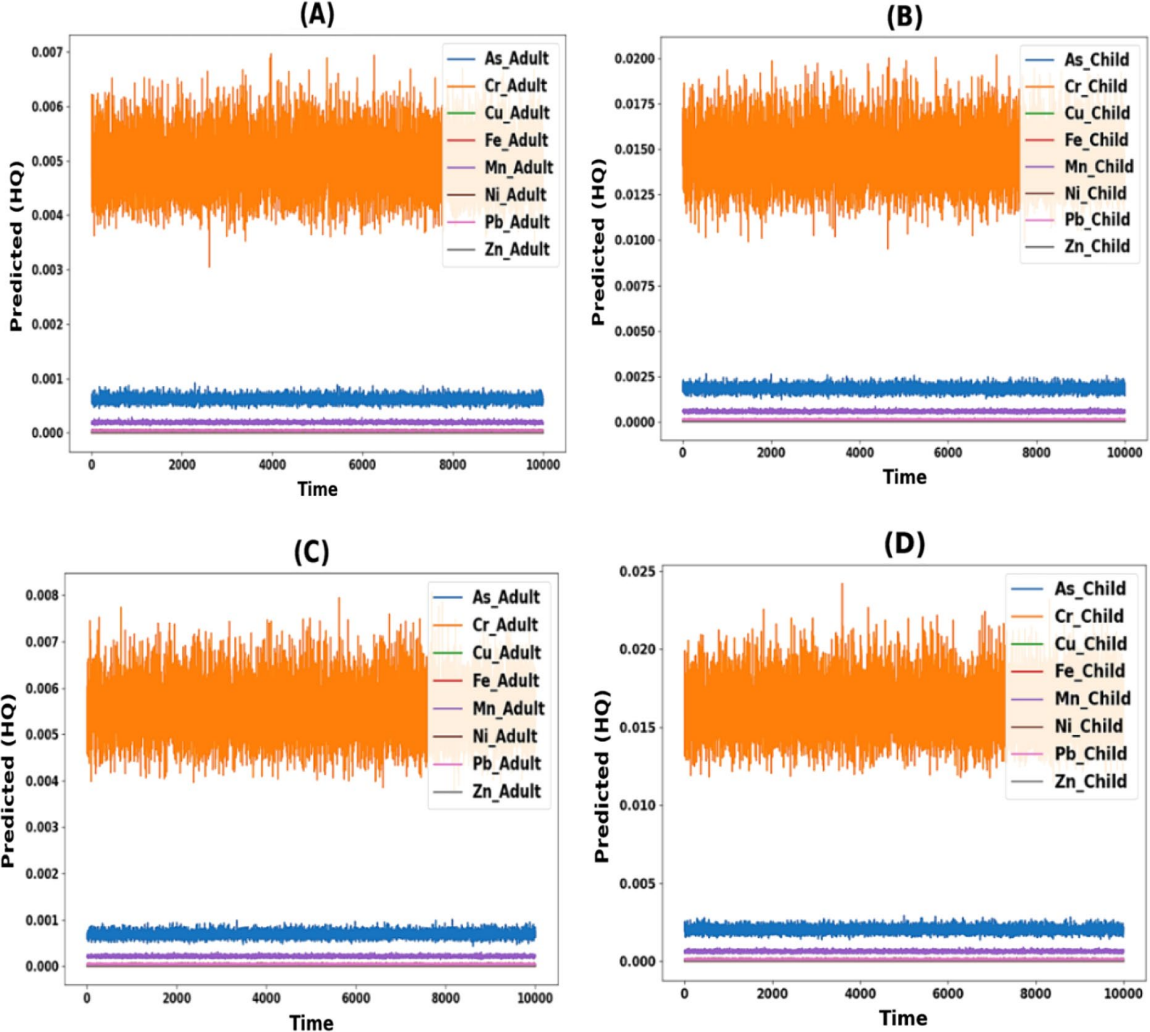
Predicted dermal hazard quotient **A** Adults dermal during April–September; **B** Children dermal during April–September; **C** Adults dermal during October–March & **D** Children dermal during October–March

The results of the Monte Carlo simulation indicated that the predicted HQ values for all heavy metals did not exceed the standard limits (HQ < 1). The estimated exposure levels are unlikely to pose a significant health risk for either adult or child in oral and dermal contact pathways. However, it is essential to note that risk assessments are typically based on conservative assumptions and uncertainties in the available data, therefore, it is essential to continue to monitor exposure levels and update risk assessments as new information becomes available (Fig. 10 and 11).

#### Carcinogenic health risk

In this study, the Monte Carlo method was utilized to simulate carcinogenic risk assessments obtained through the deterministic approach for both oral and dermal exposure pathways in adults and children. The 5th and 95th percentile risk exposures represented the best-case and worst-case scenarios, respectively.

##### Arsenic

**Fig. 12:**
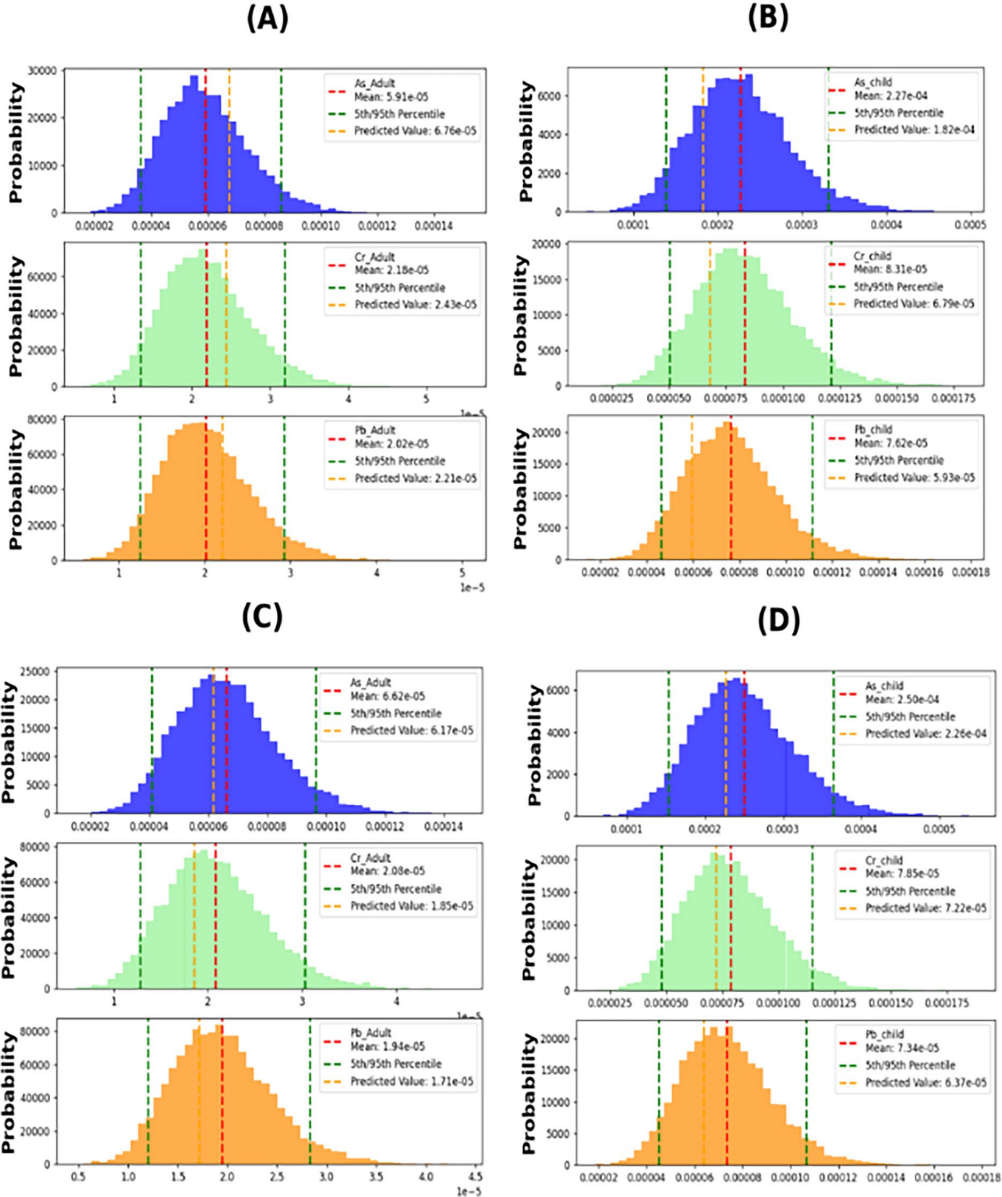
Carcinogenic risk oral: **A** adults in April–September; **B** children in April–September; **C** adults in October–March; **D** children in October–March

**Fig. 13:**
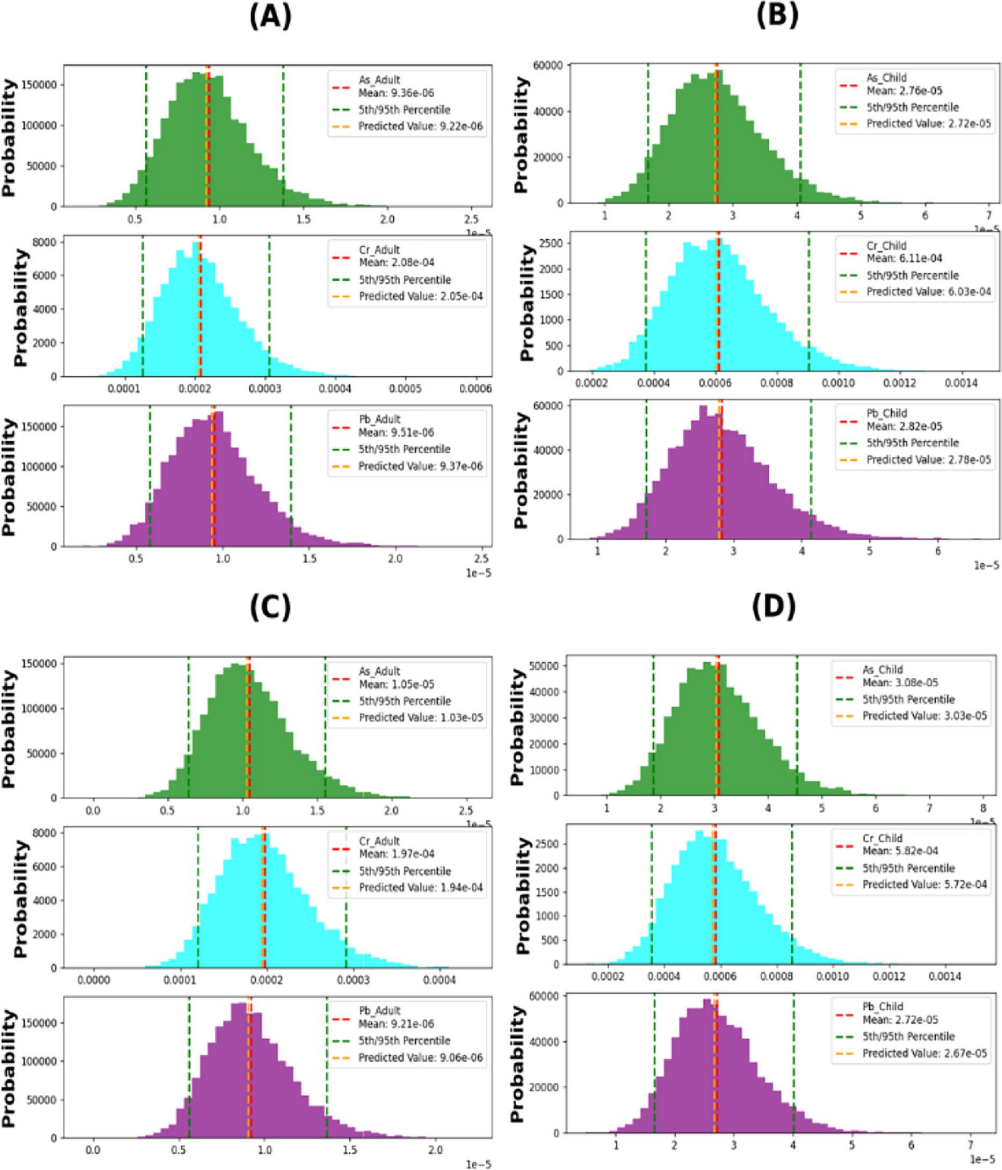
Carcinogenic risk dermal: **A** adults in April–September; **B** children in April–September; **C** adults in October–March; **D** children in October–March

The analysis of probabilities (Fig. 12 and 13A–D) suggests that the CR oral measurements in the considered population groups follow the pattern of children > adults. The findings demonstrate that during both October–March and April–September, the 95th percentile CR values in children were 3.6E−04 and 3.3E−04, respectively, while in adults, they were 9.65E−05 and 8.636E−05, respectively. These results indicate that children have a higher potential health risk, with the highest 95th percentile among the exposed groups (3.6E−04) during October–March. Additionally, the CR dermal measurements show that the 95th percentile values in children were 4.537E−05 and 4.055E−05, respectively, while in adults, they were 1.55E−05 and 1.380E−05, respectively. The overall carcinogenic risk levels in children were much higher than the acceptable cancer risk level (1.0E- 04), indicating the potential development of cancer for children in the future due to prolonged exposure to arsenic within the entire watershed. One possible explanation for the higher CR in children is their lower weight (Fallahzadeh et al., 2019). Arsenic is the primary carcinogenic substance derived from various sources in the environment, although its concentration is typically low in natural settings. Consequently, it can be deduced that human activities predominantly contribute to the presence of arsenic in surface water. These activities encompass arsenic and arsenic-containing metal mining, the utilization of arsenic and arsenide as raw materials, and coal combustion (Zhang et al., 2022).

##### Chromium

Figures 12 and 13A–D depict histograms simulating the carcinogenic risk (CR) from exposure to chromium through oral and dermal pathways in both adults and children. The CR oral results indicate that children are more vulnerable to cancer development than adults, as their oral CR levels exceed the threshold value of 1.0E−04 (oral CR 95% = 1.2E−04), whereas the CR results for adults during April–September and October–March were negligible. On the other hand, the CR dermal results for the 95th percentile confirm that both children and adults are susceptible to cancer. During April–September and October–March, the CR dermal results for children were 9.03E−04 and 8.53E−04, respectively, while the results for adults were 3.050E−04 and 2.92E−04, respectively. Therefore, reducing the environmental chromium levels is likely to decrease the associated health risks from exposure (Fallahzadeh et al., 2019).

##### Lead

Histograms in Figs. 12 and 13A–D were generated using 10,000 iterations to model the cancer risk of lead through oral and dermal exposure in adults and children. The data indicate that children are at a higher risk of developing cancer compared to adults, as their oral CR levels surpass the threshold value of 1.0E−04 (oral CR 95% = 1.1E−04) during April–September. However, neither children nor adults were vulnerable to cancer during October–March, with negligible results recorded for children (CR = 2.922E−05) and adults (2.922E−05 and 2.818E−05) during April–September and October–March, respectively. Nonetheless, the 95th percentile results of CR dermal demonstrate that neither adults nor children were susceptible to cancer. The CR dermal results for children during April–September and October–March were 4.134E−05 and 4.004E−05, respectively, while the results for adults were 1.400E−05 and 1.363E−05, respectively. Therefore, reducing lead levels in the environment is crucial in mitigating the associated health risks, especially for children.

#### Sensitivity analysis

**Fig. 14:**
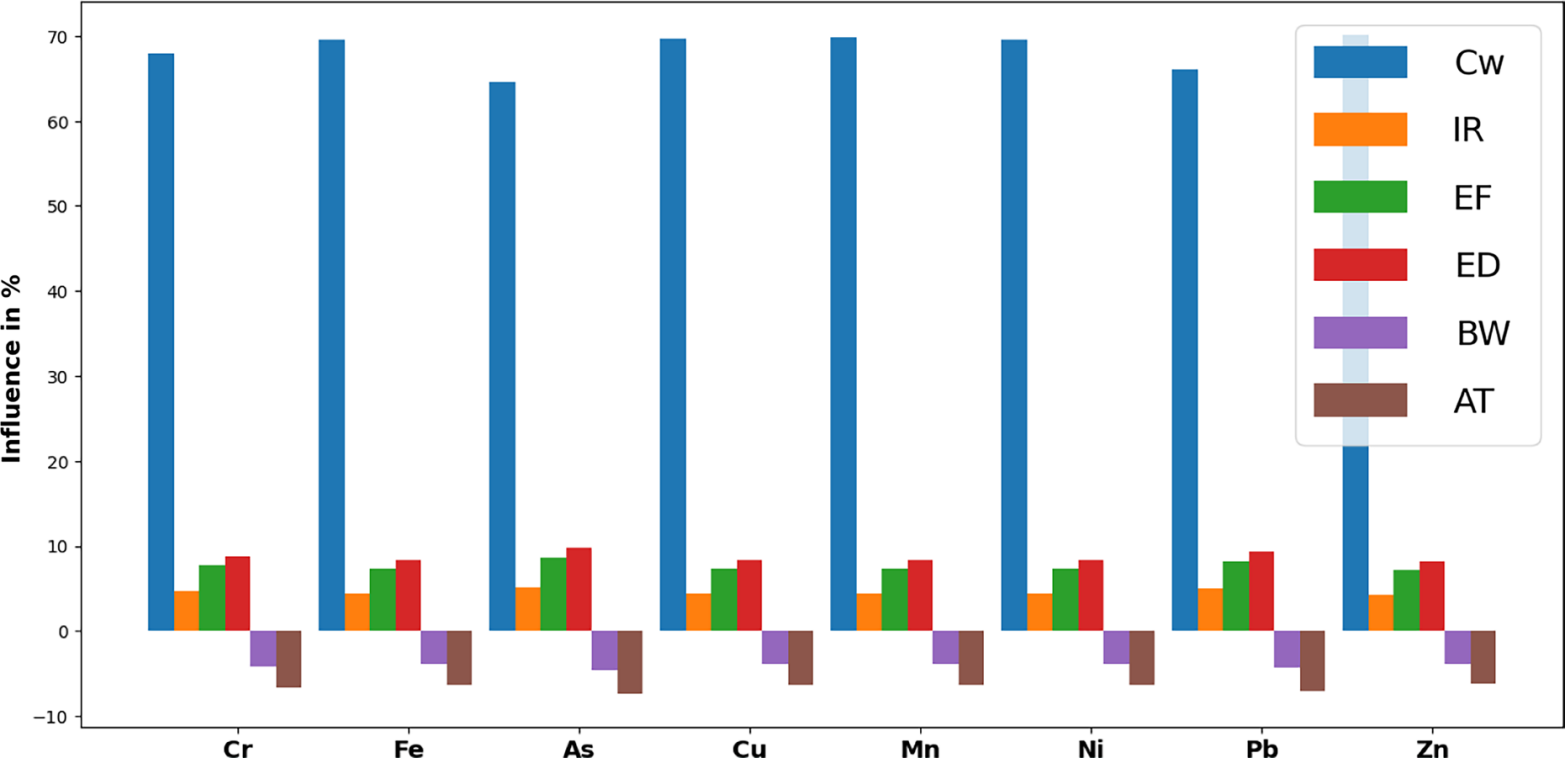
Bar plot of the sensitivity analysis

It’s important to highlight that elevated concentrations of heavy metals in surface water can lead to interactive effects on human health that should not be ignored. Therefore, we conducted a sensitivity analysis to identify the most significant parameters contributing to health risks. As depicted in Fig. 14, the parameters with the most notable influence on increasing the risk of carcinogenicity due to the consumption of trace metals in domestic water were ranked in descending order as follows: concentration of metal (Cw) > ingestion rate of water (IR) > exposure frequency (EF) > exposure duration (ED) > average time (AT) > body weight (BW) (Fallahzadeh et al., 2019). Notably, the sensitivity analysis illustrated an inverse relationship between body weight and sensitivity, indicating that higher body weight leads to reduced sensitivity (Ghoochani et al., 2019). The presence of heavy metal elements has been observed to positively impact exposure frequency and ingestion rate, while concurrently having a negative effect on body weight. Moreover, a higher output value is associated with an increased risk value, signifying a more substantial impact (Jiang et al., 2021). Therefore, implementing stringent controls to regulate heavy metal levels in drinking water systems is crucial. This can be achieved through routine monitoring of heavy metals, with a particular focus on chromium concentrations. These findings align with prior research on human health risk assessment (Mahato & Gupta, 2022). However, while sensitivity analysis focused on individual elements can yield valuable insights, it’s vital to consider the potential combined effects of these contaminants on human health. Therefore, it is recommended that future studies integrate sensitivity analyses of multiple contaminants to gain a more comprehensive understanding of their collective impact on human health. Such an approach will provide a solid foundation for crafting risk management strategies and ensuring effective protection of public health from exposure to these hazardous substances.

## Conclusion

The primary objective of this research was to investigate the levels of specific metals in the water of the Danube River in Hungary and assess their potential environmental and human health risks. The concentrations of manganese in both the southern (Hercegszántó and Hercegszántó cities) and northern (Dunaföldvár and Dunaföldvár cities) parts of the study area exceeded permissible drinking water limits. Iron concentrations also exceeded acceptable levels at all locations. This consistent increase in iron concentrations could be attributed to industrial activities releasing iron-containing effluents into the river or its tributaries. Conversely, the use of iron-based fertilizers in regional agriculture might contribute to elevated iron levels, while the selective use of manganese-based fertilizers could lead to high concentrations in specific agricultural regions. The study found no ecological risk (RI < 30) in the Danube River, as concentrations of arsenic (As), chromium (Cr), copper (Cu), nickel (Ni), lead (Pb), and zinc (Zn) remained below limit values. According to the Heavy Metal Pollution Index, all water samples collected during both periods indicated moderate pollution levels (15 < HPI < 30). However, the Metal Index revealed that the middle section of the river showed moderate pollution levels (2 < MI < 4), while the north and south parts exhibited higher pollution levels (4 < MI < 6).4

In terms of health risk assessment, non-carcinogenic health risk was evaluated using the hazard quotient (HQ) and hazard index (HI) for both oral and dermal exposures. The results indicated no significant risk for both adults and children (HI < 1 and HQ < 1). However, arsenic posed a carcinogenic health risk to children through oral exposure (CR > 1 × 10^−4^), and chromium presented a carcinogenic health risk for both adults and children through dermal exposure in both periods (CR > 1 × 10^−4^). Notably, the data suggested that children were more susceptible to lead-induced cancer compared to adults, as their oral cancer risk (CR) exceeded the threshold value of (CR > 1 × 10^−4^).

The application of the Monte Carlo simulation method proved valuable for predicting hazard quotient (HQ) for oral and dermal exposures in both adults and children, as well as simulating the carcinogenic effects of arsenic (As), chromium (Cr), and lead (Pb), thereby validating deterministic calculations. Sensitivity analysis indicated that metal concentration, intake rate, and exposure frequency were the most influential parameters contributing to the risk of carcinogenicity in the river. Therefore, regular monitoring of heavy metal concentrations in the Danube River is essential to identify any potential increases resulting from human activities. Moreover, proper treatment of wastewater from industrial sectors before discharge into the river is crucial to ensuring compliance with standard limits.

### Supplementary Information

Below is the link to the electronic supplementary material.

Supplementary file1 (DOCX 42 kb)
