# Correction: Investigating the impacts of heavy metal(loid)s on ecology and human health in the lower basin of Hungary’s Danube River: A Python and Monte Carlo simulation-based study

**DOI:** 10.1007/s10653-023-01777-4

**Published:** 2023-11-17

**Authors:** Omar Saeed, András Székács, Győző Jordán, Mária Mörtl, Mostafa R. Abukhadra, Mohamed Hamdy Eid

**Affiliations:** 1 Doctoral School of Environmental Science, Hungarian University of Agriculture and Life Sciences (MATE), Páter Károly u. 1, Gödöllő, 2100 Hungary; 2 Agro-Environmental Research Centre, Institute of Environmental Sciences, Hungarian University of Agriculture and Life Sciences, Herman Ottó út 15, Budapest, H-1022 Hungary; 3 Eötvös Loránd University (ELTE), Budapest, Hungary; 4 Geology Department, Faculty of Science, Beni-Suef University, Beni-Suef, 65211 Egypt; 5 Institute of Environmental Management, Faculty of Earth Science, University of Miskolc, Miskolc, 3515 Hungary

**Correction: Environ Geochem Health** 10.1007/s10653-023-01769-4

In this article, the statement in the Funding information section was incorrectly given as 'Open access funding provided by Hungarian University of Agriculture and Life Sciences. The authors have not disclosed any funding' and should have read ''Open access funding provided by Hungarian University of Agriculture and Life Sciences. This research was funded by Hungarian National Research, Development, and Innovation Office, Grant Number TKP2021-NVA-22'.

The original article has been corrected.

