# Supplementary material for: Investigating the impacts of heavy metal(loid)s on ecology and human health in the lower basin of Hungary’s Danube River: A Python and Monte Carlo simulation-based study
Source: Environ Geochem Health. 2023 Oct 16;45(12):9757–84. doi: 10.1007/s10653-023-01769-4 (PMC10673977; doi:10.1007/s10653-023-01769-4)
Supplement: Supplementary file 1 — Supplementary file1 (DOCX 42 kb) [file 10653_2023_1769_MOESM1_ESM.docx]

**Investigating the Impacts of Heavy Metal (Loid) on Ecology and Human Health in the Lower Basin of Hungary's Danube River: A Python and Monte Carlo Simulation-based Study**

**Table.S1** The parameters for the computation of HQ, HI, RI and CR

| **HM** | **As** | **Cr** | **Cu** | **Fe** | **Mn** | **Ni** | **Pb** | **Zn** | **Ref.** |
| --- | --- | --- | --- | --- | --- | --- | --- | --- | --- |
| **RfD Oral(mg/kg/day)** | 0.0003 | 0.003 | 0.04 | 0.7 | 0.024 | 0.02 | 0.0014 | 0.3 | (Xu et al. 2020) |
| **ABS** | 1 | 0.025 | 0.3 | 0.2 | 0.04 | 0.04 | 0.3 | 0.2 | (Xu et al. 2020) |
| **Rfd Dermal(mg/kg/day)** | 0.0003 | 0.000075 | 0.012 | 0.14 | 0.00096 | 0.0008 | 0.00042 | 0.06 | (Xu et al. 2020) |
| **CSFing mg/kg/day** | 1.5 | 0.5 |  |  |  |  | 0.5 |  | (Xu et al. 2020) |
| **CSFderm** | 50 | 500 |  |  |  |  | 500 |  | (Xu et al. 2020) |
| **Kp** | 0.001 | 0.002 | 0.001 | 0.001 | 0.001 | 0.0002 | 0.0001 | 0.0006 | (USEPA 2004) |
| **Background (µg/g)** | 10 | 30 | 30 | 15000 | 500 | 20 | 20 | 100 | (Woitke et al. 2003) |
| **Tr** | 10 | 2 | 5 | 1 | 1 | 5 | 5 | 1 | (Hakanson 1980) |

**Table.S2** The reported toxic metals in the lower Danube River and other global rivers in µg.L^-1^

| Sampling area/Country | Survey period | | Heavy metals | | | | | | | | | Ref. |
| --- | --- | --- | --- | --- | --- | --- | --- | --- | --- | --- | --- | --- |
|  |  | | **Cr** | | **Pb** | | **Cu** | **Zn** | **Fe** | **Ni** | **As** |  |
| Holbina-Dunavat/RO | 2007 | | _ | | 42.6 | | – | 334 | – | 86 | – | (Burada et al. 2014) |
| Sontea-Fortuna/RO |  |  | __ | | 21 | | – | 144 | – | 104 | – |  |
| Matita-Merhei/RO |  |  | _ | | 24 | | – | 231 | – | 68 | – |  |
| Somova-Parches/RO |  |  | _ | | 9.3 | | – | 176 | – | 64 | – |  |
| Holbina-Dunavat/RO | 2008 | | _ | | 48 | | – | 249.6 | – | 38 | – |  |
| Sontea-Fortuna/RO |  |  | _ | | 34.7 | | – | 124 | – | 32 | – |  |
| Matita-Merhei/RO |  |  | _ | | 36 | | – | 165 | – | 27 | – |  |
| Somova-Parches/RO |  |  | _ | | 8 | | – | 168 | – | 48 | – |  |
| Holbina-Dunavat/RO | 2009 | | _ | | 32 | | – | 311 | – | 41 | – |  |
| Sontea-Fortuna/RO |  |  | _ | | 28 | | – | 178.9 | – | 88 | – |  |
| Matita-Merhei/RO |  |  | _ | | 29 | | – | 187 | – | 39 | – |  |
| Somova-Parches/RO |  |  | _ | | 6.8 | | – | 128 | – | 36 | – |  |
| Holbina-Dunavat/RO | 2010 | | _ | | 36 | | – | 312 | – | 98 | – |  |
| Sontea-Fortuna/RO |  |  | _ | | 11 | | – | 197 | – | 92 | – |  |
| Matita-Merhei/RO |  |  | _ | | 23 | | – | 182.2 | – | 89 | – |  |
| Somova-Parches/RO |  |  | _ | | 7 | | – | 126 | – | 49 | – |  |
| Holbina-Dunavat/RO | 2011 | | _ | | 39 | | – | 209 | – | 55 | – |  |
| Sontea-Fortuna/RO |  |  | _ | | 17 | | – | 172 | – | 41 | – |  |
| Matita-Merhei/RO |  |  | _ | | 32 | | – | 161 | – | 85 | – |  |
| Somova-Parches/RO |  |  | _ | | 6.9 | | – | 151 | – | 48 | – |  |
| Somova Lake/RO | 2007–2012 Spring | | _ | | 7.7–11 | | – | 161–209.8 | – | 49–78.9 | – | (Burada et al.) |
| Rotundu Lake/RO |  |  | _ | | 6.8–8.9 | | – | 124–181 | – | 40–59 | – |  |
| Somova Lake/RO | 2007–2012 Summer | | _ | | 6.1–7.5 | | – | 138–188.7 | – | 41–59 | – |  |
| Rotundu Lake/RO |  |  | _ | | 6.2–7.2 | | – | 100–164 | – | 27.9–46.6 | – |  |
| Somova Lake/RO | 2007–2012  Autumn | | _ | | 6.8–8.9 | | – | 147–179.6 | – | 43–71 | – |  |
| Rotundu Lake/RO |  |  | _ | | 6.2–7.8 | | – | 117.7–179.3 | – | 36–52.8 | – |  |
| Babadag Lake/RO | 2008 | June | | _ | | 1.6 | – | – | – | 2 | 2 | (Rusu et al. 2014) |
|  |  | July | | _ | | 1.5 | – | – | – | 2.1 | 2.1 |  |
|  |  | September | | _ | | 1.7 | – | – | – | 2.3 | 1.9 |  |
|  | 2009 | June | | _ | | 1.5 | – | 1.2 | – | 2.1 | 2.2 |  |
|  |  | July | | _ | | 1.5 | – | 1.3 | – | 2.1 | 2.4 |  |
|  |  | September | | _ | | 1.5 | – | 1.1 | – | 2.3 | 2.3 |  |
|  | 2010 | June | | _ | | 1.5 | – | 1.2 | – | 2 | 2.9 |  |
|  |  | July | | _ | | 1.5 | – | 1.3 | – | 2.1 | 2.8 |  |
|  |  | September | | _ | | 1.5 | – | 1.1 | – | 2.3 | 2.7 |  |
| Galati/RO | 2010 | August | | _ | | 21.4 ± 1.6 | 112.3 ± 3.8 | 47.1 ± 2.0 | – | – | – | (Ioniţă et al. 2014) |
| Tulcea/RO |  |  |  | _ | | 14.3 ± 2.5 | 93.5 ± 2.5 | 32.5 ± 1.6 | – | – | – |  |
| Novi Sad/SE | 2011 | June | | _ | | 0.6 | 6 | 20 | 260 | 1.4 | 1 | (Milošković et al. 2016) |
| Zemun/SE | 2013 | October | | _ | | 0.5 | 6 | 20 | 210 | 3 | 0.6 |  |
| Radujevac/SE |  |  |  | _ | | 0.5 | 6 | 9 | 80 | 0.9 | 2 |  |
| Coronini,Caras-Severin/RO | 2010 December – 2012 July | | _ | | _ | | 1.46 ± 0.59 | 0.78 ± 0.86 | – | – | – | (Matache et al. 2013) |
| Bazias/RO |  |  | _ | | _ | | 1.89 ± 0.04 | 1.8 ± 3.49 | – | – | – |  |
| Divici/RO |  |  | – | | – | | 3.17 ± 1.86 | 1.35 ± 1.89 | – | – | – |  |
| Batajnica, Belgrade/SE | 2012 | Autumn | | _ | | ND | 4 ± 1 | 32 ± 2 | 330 |  |  | (Milanov et al. 2016) |
| Xiangxi River, China | 2021 |  | | _ | | 10.59 ± 7.33 | 3.21 ± 0.83 | 2.4 ± 2.61 |  | 6.2 ± 1.66 | 0.65 ± 0.46 | (Wang et al. 2015) |

**References**

Burada A, Topa CM, Georgescu LP, et al Heavy Metals Environment Accumulation in Somova—Parches Aquatic Complex from the Danube Delta Area. bch.ro

Burada A, Ţopa CM, Georgescu LP, et al (2014) AACL BIOFLUX Heavy metals accumulation in plankton and water of four aquatic complexes from Danube Delta area 1,2. 7:

Hakanson L (1980) An ecological risk index for aquatic pollution control.a sedimentological approach. Water Res 14:975–1001. https://doi.org/10.1016/0043-1354(80)90143-8

Ioniţă C, Mititelu M, Moroşan E (2014) ANALYSIS OF HEAVY METALS AND ORGANIC POLLUTANTS FROM SOME DANUBE RIVER FISHES. Farmacia 62:

Matache ML, Marin C, Rozylowicz L, Tudorache A (2013) Plants accumulating heavy metals in the Danube River wetlands. J Environ Heal Sci Eng 11:1–7. https://doi.org/10.1186/2052-336X-11-39/FIGURES/2

Milanov DR, Krstić PM, Marković VR, et al (2016) Analysis of heavy metals concentration in tissues of three different fish species included in human diet from Danube river, in the Belgrade region, Serbia. Acta Vet 66:89–102. https://doi.org/10.1515/ACVE-2016-0007

Milošković A, Dojčinović B, Kovačević S, et al (2016) Spatial monitoring of heavy metals in the inland waters of Serbia: a multispecies approach based on commercial fish. Environ Sci Pollut Res 23:9918–9933. https://doi.org/10.1007/S11356-016-6207-2/FIGURES/5

Rusu T, Rusu T, Vida C, et al (2014) Determining Heavy Metal Concentrations in the Waters of Babadag River. Citeseer

USEPA (2004) Risk assessment guidance for superfund (RAGS). Volume I. Human health evaluation manual (HHEM). Part E. Supplemental guidance for dermal risk assessment. Us Epa 1:. https://doi.org/EPA/540/1-89/002

Wang Y, Yang L, Kong L, et al (2015) Spatial distribution, ecological risk assessment and source identification for heavy metals in surface sediments from Dongping Lake, Shandong, East China. CATENA 125:200–205. https://doi.org/10.1016/J.CATENA.2014.10.023

Woitke P, Wellmitz J, Helm D, et al (2003) Analysis and assessment of heavy metal pollution in suspended solids and sediments of the river Danube. Chemosphere 51:633–642. https://doi.org/10.1016/S0045-6535(03)00217-0

Xu J, Zheng L, Xu L, et al (2020) Identification of dissolved metal contamination of major rivers in the southeastern hilly area, China: distribution, source apportionment, and health risk assessment. Environ Sci Pollut Res 27:3908–3922. https://doi.org/10.1007/s11356-019-06774-8
